# Modulation of SIRT1/PPARγ pathways and tight junction proteins by nicotinamide riboside under chronic variable stress

**DOI:** 10.1007/s13105-026-01153-7

**Published:** 2026-01-22

**Authors:** Abdullah Celik, Nurhan Sahin, Cemal Orhan, Besir Er, Fusun Erten, Busra Ozmen, Mehmet Tuzcu, Ibrahim Hanifi Ozercan, Kazim Sahin

**Affiliations:** 1 Department of Animal Nutrition, Faculty of Veterinary Medicine, Firat University, Elazig, 23119 Türkiye; 2 Department of Medical Services and Techniques, Vocational School of Health Services, Firat University, Elazig, 23119 Türkiye; 3 Department of Veterinary Science, Pertek Vocational School, Munzur University, Tunceli, 62500 Türkiye; 4 Department of Biology, Faculty of Science, Firat University, Elazig, 23119 Türkiye; 5 Department of Pathology, Medicine Faculty, Health Sciences Institution, Firat University, Elazig, 23119 Türkiye

**Keywords:** Stress, Nicotinamide riboside, Liver, Gut

## Abstract

**Abstract:**

Chronic stress disrupts homeostasis, leading to major health problems such as liver damage, intestinal barrier dysfunction, and impaired glucose metabolism. Although current treatments, including anxiolytics, sedatives, antidepressants, and beta blockers, are effective, their adverse effects emphasize the need for safer alternatives. Nicotinamide riboside (NR), a precursor of nicotinamide adenine dinucleotide (NAD+), plays a central role in energy metabolism and oxidative stress regulation; elevated NAD + levels have been associated with reduced risk of chronic diseases such as obesity and type 2 diabetes. However, the effects of NR on liver metabolism, intestinal barrier integrity, and related protein pathways remain unclear. This study investigated the effects of NR supplementation in rats exposed to chronic variable stress (CVS). Fifty-six male Sprague Dawley rats were divided into normal and CVS groups and treated in a 2 × 4 factorial design with 0, 150, 300, or 600 mg/kg NR. Under CVS conditions, serum glucose, corticosterone, ACTH, and insulin levels increased, whereas NAD+, NADPH, nicotinamide (NAM), and nicotinic acid (NA) decreased significantly (*p* < 0.001). NR supplementation effectively corrected these biochemical imbalances and upregulated hepatic markers, including PPARγ, SIRT1, GLUT2, IRS1, and FASN (*p* < 0.001). Furthermore, the increased expression of key transport proteins such as PepT1, LAT2, EAAT3, FABP2, and FATP4 contributed to maintaining intestinal barrier integrity and improving gut health. NR also promoted the recovery of tight and adherens junction proteins. Notably, high-dose NR (600 mg/kg) markedly alleviated liver fibrosis, improved glucose metabolism, and strengthened intestinal barrier function, demonstrating its therapeutic potential as an alternative strategy against stress-induced metabolic disorders.

**Key points:**

• *NR mitigated chronic stress-induced liver, intestinal, and glucose dysregulation*.

• *NR improved glycemia and NAD⁺-related biomarkers under stress*.

• *NR reduced hepatic fibrosis markers*.

• *NR strengthened TJ/AJ proteins, supporting intestinal barrier integrity*.

• *Findings support NR’s therapeutic potential in stress-related metabolism*.

**Supplementary Information:**

The online version contains supplementary material available at 10.1007/s13105-026-01153-7.

## Introduction

Stress is defined as a physiological or psychological threat that disrupts homeostatic balance and contributes to metabolic, endocrine, immune, and structural dysfunction across multiple organ systems. Chronic stress is now recognized as a major global health burden associated with metabolic disease, intestinal disorders, neuropsychiatric conditions, and systemic inflammation [1–3]. Experimental models that capture the complexity and unpredictability of human stress exposure are essential for understanding these pathophysiological outcomes. The chronic variable stress (CVS) paradigm differs fundamentally from single-stressor or predictable stress models in that animals are exposed to alternating, unpredictable stressors, preventing habituation and producing cumulative multisystem disturbances closely resembling real-life human stress patterns [4–6]. CVS induces persistent activation of the hypothalamic–pituitary–adrenal axis, sustained glucocorticoid elevation, mitochondrial dysfunction, oxidative and inflammatory activation, intestinal barrier breakdown, dysbiosis, and metabolic impairment in both the liver and intestine [7–10]. These multifaceted disturbances make CVS a uniquely valuable system for evaluating therapeutic candidates that target metabolic, epithelial, redox, and neuroendocrine pathways simultaneously.

Because CVS induces parallel disruptions in hepatic and intestinal pathways, the biomarkers selected in this study were chosen to represent an integrated liver–gut metabolic axis rather than independent endpoints. CVS suppresses glucose transport and insulin signaling by downregulating GLUT2 and IRS1, which together form the core glucose–insulin regulatory axis and are rapidly impaired under chronic glucocorticoid overload [11, 12]. CVS also activates the FASN lipogenic pathway, which is strongly induced by oxidative and endoplasmic reticulum stress and leads to hepatic triglyceride accumulation and steatosis [13, 14]. In the intestine, PepT1, LAT2, and EAAT3 regulate peptide and amino acid transport, mTOR signaling, and incretin-mediated insulin regulation; all are sensitive to stress-induced epithelial atrophy and inflammation [15, 16]. In parallel, FABP2 and FATP4 mediate dietary fatty acid absorption and intracellular trafficking, and disruption of these proteins contributes to systemic inflammation, lipid malabsorption, and insulin resistance [17, 18]. Collectively, changes in these biomarkers provide a mechanistic map of glucose regulation, lipid synthesis, amino acid transport, and intestinal absorptive function, pathways known to be simultaneously impaired under CVS, thereby enabling an integrated evaluation of both stress pathology and potential therapeutic effects.

Nicotinamide riboside (NR), a vitamin B3 derivative, is a precursor of nicotinamide adenine dinucleotide (NAD⁺), an essential cofactor for mitochondrial energy metabolism, DNA repair, and cellular stress responses [19]. Its strong NAD⁺-enhancing capacity and favorable safety profile have contributed to growing interest in its therapeutic use [20]. NAD⁺ plays a central role in cellular homeostasis by supporting energy production, genomic stability, and the activity of NAD⁺-dependent enzymes such as SIRT1 and PARPs [21]. Reduced NAD⁺ levels impair mitochondrial function, limit DNA repair capacity, and compromise cellular defense mechanisms [22]. Studies have shown that NR supplementation elevates NAD⁺ levels and produces beneficial effects in conditions characterized by inflammation, oxidative stress, and metabolic dysfunction, including hepatic steatosis and diet-induced obesity [15, 17]. NR has been reported to reduce high-fat diet–induced steatosis by increasing mitochondrial fatty acid oxidation, improving SIRT1 signaling, and reducing lipid accumulation [23–26]. Moreover, NR suppresses inflammatory responses and enhances antioxidant defenses, thereby protecting cells from oxidative injury [25]. NR also supports intestinal health by maintaining tight junction integrity, reducing inflammation, modulating the gut microbiota, and protecting intestinal endothelial cells from oxidative damage [27–29]. These improvements in barrier function help prevent bacterial translocation and reduce systemic inflammatory burden [30, 31]. Together, these findings provide a strong rationale for investigating NR as a therapeutic candidate for stress-related metabolic and intestinal disorders. However, recent findings indicate that NR’s biological effects may be context dependent under conditions of sustained stress. In aged mice, NR supplementation altered hematopoietic dynamics and increased stress sensitivity and anxiety-like behaviors, suggesting a bidirectional relationship with neuroendocrine pathways [32]. In a chronic corticosterone exposure model, NR and phycocyanin oligopeptides jointly modulated mitochondrial activity, oxidative stress, and behavioral stress susceptibility, indicating that NR can influence both vulnerability and resilience pathways under persistent glucocorticoid load [33]. These emerging observations highlight the need to examine NR within a multifactorial stress framework rather than relying exclusively on metabolic or aging models.

CVS is particularly appropriate for such investigation because it reproduces the unpredictable, diverse, and cumulative nature of human stress exposure. CVS simultaneously induces hepatic and intestinal injury, mitochondrial dysfunction, NAD⁺ depletion, oxidative and inflammatory activation, tight junction disruption, mucus loss, and suppression of nutrient transporters—pathways that directly overlap with those modulated by NR and central to its mechanism of action [7, 30]. Thus, CVS provides a biologically rich and clinically relevant platform for assessing NR’s ability to restore NAD⁺-dependent metabolic, mitochondrial, and epithelial-protective processes under sustained stress. Although NR has been extensively studied in non-alcoholic fatty liver disease, obesity, type 2 diabetes, neurodegeneration, aging, and cancer [19, 34], its protective effects against CVS-induced hepatic and intestinal dysfunction remain largely unexplored. To address this gap, the present study investigated the molecular mechanisms underlying NR’s protective effects against CVS-induced damage, with a specific focus on glucose metabolism, intestinal barrier function, and oxidative stress. By targeting these critical pathways, the study identifies NR as a promising therapeutic agent for managing metabolic and gastrointestinal disorders associated with chronic stress.

## Materials and methods

### Experimental animals

In this study, 56 male Sprague–Dawley rats (8 weeks old, 180 ± 20 g) were housed in polypropylene cages under controlled environmental conditions (22 ± 2 °C, 55 ± 5% humidity, and a 12/12-hour light/dark cycle), with feed and water provided ad libitum. The sample size was determined as seven rats per group (*n* = 7) based on an a priori power analysis conducted with G*Power software (version 3.1.9.2), assuming an effect size of 0.70, a type I error (α) of 0.05, and a power (1–β) of 85% [35, 36]. Statistical analyses were performed using IBM SPSS Statistics software (version 22.0). All animal experiments were approved by the Local Ethics Committee for Animal Experiments of Fırat University (protocol number 27.02.2024–22557) and were conducted at the Experimental Research Center of Fırat University (FÜDAM). The design and reporting of this study adhered to the ARRIVE guidelines [37].

## Experimental design

**Fig. 1:**
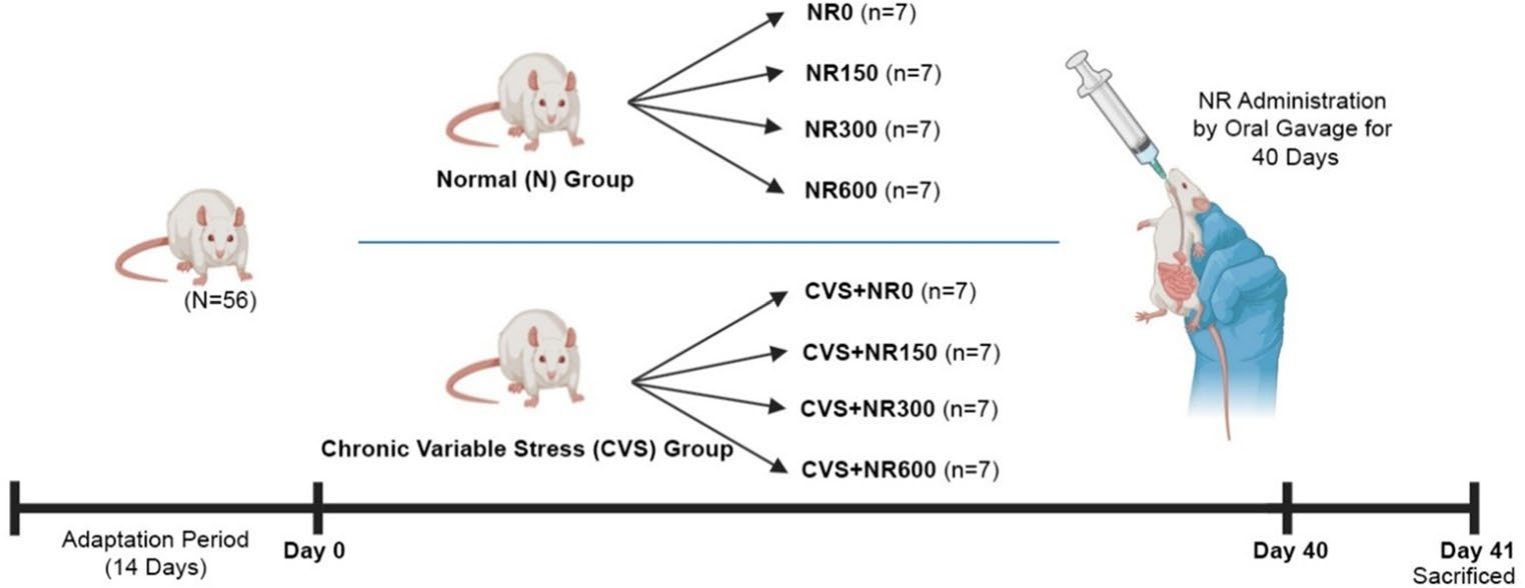
The experimental design was as follows: NR refers to Nicotinamide Riboside. NR0 represents the normal control group, NR150 the normal group receiving 150 mg/kg NR, NR300 the normal group receiving 300 mg/kg NR, and NR600 the normal group receiving 600 mg/kg NR. CVS-NR0 represents the chronic variable stress control group, while CVS-NR150, CVS-NR300, and CVS-NR600 represent the chronic variable stress groups receiving 150 mg/kg, 300 mg/kg, and 600 mg/kg NR, respectively

After a two-week adaptation period, 56 male Sprague-Dawley rats were randomly divided into eight groups (*n* = 7) in a 2 × 4 factorial design. The first factor was the housing condition (normal or stress), and the second factor was the administration of four doses of NR (0, 150, 300, and 600 mg/kg body weight) (Fig. 1). The experimental groups were as follows: a control group (NR0) consisting of rats not exposed to chronic variable stress (CVS), which were administered drinking water via oral gavage. The NR groups (NR150, NR300, and NR600) comprised rats not exposed to CVS but administered nicotinamide riboside (NR) via oral gavage at doses of 150, 300, or 600 mg/kg body weight, respectively. The stress group (CVS + NR0) included rats subjected to CVS and provided with drinking water (1 ml/animal/day). The CVS + NR groups (CVS + NR150, CVS + NR300, and CVS + NR600) consisted of rats exposed to CVS and administered via oral gavage NR at doses of 150, 300, or 600 mg/kg body weight, respectively. The study design is illustrated in Fig. 1.

The study lasted 40 days, following a two-week adaptation period. The rats were provided with a standard diet and water ad libitum throughout the experiment. The nicotinamide riboside material (Nutrition21, NY, USA; Lot No. T19269001) contained 62% active nicotinamide riboside, which is the expected specification for the NR chloride form, where the remaining fraction consists of chloride ions, hydration water, and inert excipients. This composition is consistent with previously published NR studies, and all administered doses were calculated based on the active NR content [33, 38, 39]. NR was dissolved in drinking water, prepared at the specified doses, and administered at a volume of 1 ml per animal. Oral gavage was performed daily at the same time and volume (1 ml/animal).

Since nicotinamide riboside (NR) has not previously been evaluated in any chronic variable stress (CVS) model in rats, the dose range used in this study was selected based on effective and well-tolerated doses reported in other rat disease models [23, 26, 40, 41]. This approach is widely accepted in preclinical research when stress-specific dosing data are unavailable. Previous studies have demonstrated that oral NR doses of 200 mg/kg are effective and non-toxic in chemotherapy-induced peripheral neuropathy models [42], while doses of 400 mg/kg have improved lipid metabolism, inflammation, and oxidative stress in obese Wistar rats [40, 43]. Additionally, cardiometabolic studies have successfully used NR doses ranging from 100 to 400 mg/kg, reporting significant benefits in mitochondrial function, NAD⁺ turnover, and metabolic homeostasis [26, 44]. Based on these published dose ranges, we implemented a graded NR dosing scheme (150, 300, and 600 mg/kg) to identify the most effective and biologically relevant dose in a CVS model being investigated for the first time. A structured toxicity-monitoring protocol was incorporated into the study to ensure that NR administration at 150, 300, and 600 mg/kg did not produce overt adverse effects. Throughout the 40-day experimental period, animals were monitored daily for general clinical signs including grooming behavior, locomotor activity, food intake, and fur condition.

At the end of the experiment, all the animals were decapitated by cervical dislocation, and blood, liver, and intestinal tissue samples were taken from the animals. The collected blood samples were centrifuged in gel biochemistry tubes to obtain the serum. Liver and intestinal tissue samples taken for histological examination were preserved in a 10% formaldehyde solution. For molecular analyses, tissue samples were stored in a -80 °C deep freezer (Hettich, Germany) until analysis.

## Stress model

The CVS protocol used in this study was adapted from the stress factors described by Tagliari et al. (2010) (Table 1) [2]. To prevent the rats from adapting to the stressors, a single stressor was applied at different times each day. Seven different stressors were employed, including 24-hour water restriction, cold exposure at + 4 °C for 1.5–2 h, cage placement at a 45° angle for 4–6 h, exposure to flashing light for 120–210 min (using a 40 W lamp flashing at 60 flashes per minute), movement restriction for 1–3 h using a rat restrainer, placement on a damp substrate for 1.5–2 h (300 ml of water was added to the cage to create the damp environment), and 3 days of isolation. These stressors were applied to the CVS group for 40-day period, as outlined in Table 1. In contrast, rats in the normal group were housed in a storage room without exposure to stressors throughout the study.

**Table 1:** Applied stress factors

Days	Applied Stressors
1	Restriction at 4 °C for 1.5-2 h
2	Cages placed at a 45° angle for 4–6 h
3	Flashing light for 120–210 min
4	Movement restriction for 1–3 h
5	Isolation
6	Isolation
7	Isolation
8	Housing on damp bedding for 1.5–2 h
9	Cages placed at a 45° angle for 4–6 h
10	No treatment
11	Flashing light for 120–210 min
12	Water restriction for 24 h
13	Movement restriction for 1–3 h
14	Housing on damp bedding for 1.5–2 h
15	Cages placed at a 45° angle for 4–6 h
16	Restriction at 4 °C for 1.5-2 h
17	Flashing light for 120–210 min
18	Movement restriction for 1–3 h
19	Housing on damp bedding for 1.5–2 h
20	Isolation
21	Isolation
22	Isolation
23	Restriction at 4 °C for 1.5-2 h
24	Water restriction for 24 h
25	Cages placed at a 45° angle for 4–6 h
26	Movement restriction for 1–3 h
27	Flashing light for 120–210 min
28	Movement restriction for 1–3 h
29	Housing on damp bedding for 1.5–2 h
30	No treatment
31	Water restriction for 24 h
32	Cages placed at a 45° angle for 4–6 h
33	Flashing light for 120–210 min
34	Restriction at 4 °C for 1.5-2 h
35	Isolation
36	Isolation
37	Isolation
38	Flashing light for 120–210 min
39	Housing on damp bedding for 1.5–2 h
40	Movement restriction for 1–3 h

## Biochemical analysis

Serum concentrations of glucose, triglycerides, urea, and creatinine, along with the activities of alanine aminotransferase (ALT) and aspartate aminotransferase (AST), were assessed using rat-specific kits on a biochemical autoanalyzer (LABGEOPT10V-Samsung, Labgeo, South Korea).

Serum levels of corticosterone, adrenocorticotropic hormone (ACTH), and insulin, as well as liver levels of triglyceride, NAD+, NAM, NA, and NADPH, were measured using an ELISA device (Elx-800; Bio-Tek, Vermont, USA) following the protocols of commercial kits (Corticosterone: Cat. No. 201-11-0497; ACTH: Cat. No. 201-11-0185; Insulin: Cat. No. 201-11-0708; Triglyceride: Cat. No. 201-11-0250; NAD⁺: Cat. No. 201-11-2313; NAM: Cat. No. 201-11-5145; NA: Cat. No. 201-11-5146; NADPH: Cat. No. 201-11-0699, all from SunRed Biotechnology Company, Shanghai, China).

## Western blotting analysis

Liver peroxisome proliferator-activated receptor gamma (PPARγ), glucose transporter 2 (GLUT2), insulin receptor substrate-1 (IRS-1), Sirtuin 1 (SIRT1), and fatty acid synthase (FASN) levels and jejunal tight and adherence junction-associated proteins (claudin-1, claudin-4, occludin, zonula occludens-1), MUC2, sodium-dependent glucose transporter 1 (SGLT1/SLC5A1), GLUT2, peptide transporter 1 (PepT1), L-type amino acid transporter 2 (LAT2), excitatory amino acid transporter 3 (EAAT3), fatty acid-binding protein 2 (FABP2) and fatty acid transporter protein 4 (FATP4) were determined by sodium dodecyl sulfate-polyacrylamide gel electrophoresis (SDS-PAGE) followed by Western blotting [45–47]. The biomarker panel was intentionally designed to represent four major metabolic and intestinal pathways known to be disrupted by chronic variable stress (CVS): glucose homeostasis (GLUT2, IRS1), lipid metabolism (FASN), amino acid and peptide absorption (PepT1, LAT2, EAAT3), and fatty acid handling (FABP2, FATP4). Including these markers allowed for an integrated assessment of liver-gut metabolic dysfunction and provided a mechanistic framework for evaluating the restorative effects of NR under CVS conditions. Tissue samples were mechanically homogenized (three times for 10 s each) for 30 s in radioimmunoprecipitation assay buffer (1:10, w/v) containing a protease inhibitor cocktail (Sigma, St. Louis, MO, USA), and the homogenates were centrifuged for 60 min at 15,000 rpm at 4 °C. Determination of protein content in the supernatants was carried out using a microvolume spectrophotometer (MaestroNano, Maestrogen Inc., USA). Homogenates were mixed 1:1 with 2x Laemmli buffer, heated at 95 °C for 5 min, separated on 12% SDS–PAGE, and transferred to nitrocellulose membranes using a semi-dry system (Power Blotter, Thermo Fisher, Waltham, USA). Membranes were blocked with 5% bovine serum albumin (BSA) for 2 h at room temperature and incubated overnight at 4 °C with primary antibodies as follows: PPARγ (sc-271392), GLUT2 (sc-518022), IRS-1 (sc-8038), SIRT1 (sc-74465), FASN (sc-48357), claudin-1 (sc-166338), claudin-4 (sc-376643), occludin (sc-133256), ZO-1 (sc-33725), MUC2 (sc-53381), PepT1 (sc-373742), LAT2 (sc-293242), FABP2 (sc-374482), FATP4 (sc-393309) and β-Actin (sc-517582) (all from Santa Cruz Biotechnology); SGLT1 (DF7202-100; Affinity Biosciences); EAAT3 (orb1161904; Biorbyt Ltd., Cambridge, UK). The secondary antibody was horseradish peroxidase (HRP)-conjugated anti-rabbit IgG (ab97023, Abcam, Cambridge, UK; 1:5000, 2 h, room temperature). The presence of proteins was checked using an anti-β-actin antibody (Sigma, St. Louis, MO, USA). Immunoreactive bands were visualized with diaminobenzidine (DAB), and densitometry was performed using ImageJ (NIH, Bethesda, MD, USA).

### Histopathological analysis

To evaluate the effects of NR on liver and intestinal histopathology, the tissue samples were fixed in 10% formalin and processed using a standard protocol involving dehydration with graded alcohol, clearing with xylene, and embedding in paraffin. The tissues were sectioned into 5-µm thick slices using a microtome for further analysis. The sections were stained with Hematoxylin and Eosin (H&E) and examined under a light microscope. Intestinal villus height was measured from the apex of the villus to the lamina propria, while crypt depth was assessed accordingly [48, 49].

### Statistical analysis

Data are expressed as mean ± SEM. A two-way analysis of variance (Two-way ANOVA) was conducted using the General Linear Model procedure to evaluate the data. Linear, quadratic, and cubic responses to NR levels were obtained using polynomial contrasts. The effects of condition (normal vs. stress), dose effects of the administered substances, and interactions between the condition and dose were determined. The following linear model was applied: yij = µ + Si + MgTDj + (S*MgTD)ij + eij, where y represents the response variable, µ is the population mean, S denotes the main effect of stress (environment), NRD represents the main effect of NR levels, S*NRD refers to the interaction effect between stress and NR levels, and e is the residual error [N (σ, µ; 0, 1)]. A polynomial contrast analysis was applied to determine the nature of the response to increasing NR levels. A p-value of < 0.05 was considered statistically significant.

## Results

### Body weight

**Fig. 2:**
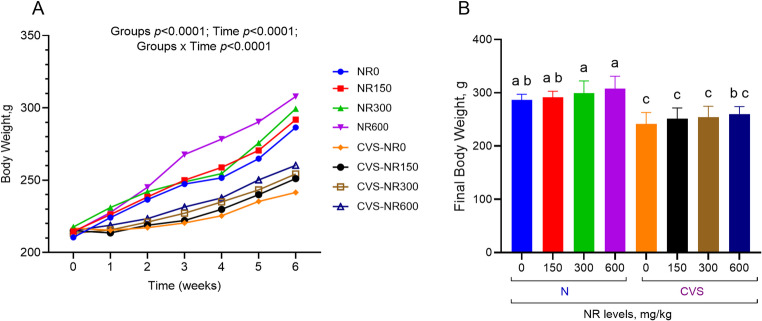
Weekly body weight changes (**A**) and final body weight (**B**) in normal and chronic variable stress (CVS)-exposed rats with different doses of nicotinamide riboside (NR) supplementation. Data are expressed as mean ± SEM. Different letters (a–c) indicate statistically significant differences between groups (*p* < 0.05) according to Tukey’s post hoc test following two-way ANOVA. Two-way ANOVA revealed significant effects for body weight changes (Groups *p* < 0.0001; Time *p* < 0.0001; Groups x Time *p* < 0.0001), and final body weight (Condition *p* < 0.0001; NR *p* = 0.0454; Condition x NR *p* < 0.9700). In addition, comprehensive statistical data can be found in Supplementary Table A.1

Rats housed under normal conditions exhibited significantly greater body weight gain compared to those under CVS conditions (*p* < 0.001, Fig. 2). NR supplementation showed a linear increase in body weight gain in both the N and CVS groups (*p* < 0.05). There was no interaction between CVS and NR supplementation on body weight gain (*p* = 0.506).

### Biochemical parameters

Serum glucose, ALT, AST, corticosterone, and ACTH concentrations were significantly higher in the groups exposed to CVS factors compared to the N group (*p* < 0.001, Table 2). Specifically, in the CVS group, increasing doses of NR supplementation led to a linear decrease in these levels, with reductions of approximately 35% in glucose, 20% in ALT, %20 in AST, 48% in corticosterone, and 35% in ACTH (*p* < 0.001); however, no significant difference was observed in the normal group (*p* > 0.05, Table 2). Additionally, an interaction was observed between stress conditions and NR supplementation concerning glucose, ALT, corticosterone, and ACTH levels (*p* < 0.001). Serum triglyceride levels were found to increase in rats exposed to stressors (*p* < 0.001, Table 2). In the CVS group, a linear reduction of approximately 18% in triglyceride levels was detected with increasing doses of NR supplementation (*p* < 0.001). An interaction was also observed between stress conditions and NR supplementation on triglyceride levels (*p* < 0.05). No significant differences were observed in serum BUN or creatinine levels between the normal and CVS groups (*p* > 0.05, Table 2). Insulin levels decreased in the CVS group (*p* < 0.001, Table 2), with a significant interaction between stress and NR supplementation (*p* < 0.001).

**Table 2:** The statistical analysis of the effect of different doses of nicotinamide riboside (NR) on biochemical parameters in normal (N) and chronic variable stress (CVS)-exposed rats (Two-way ANOVA and polynomial contrast, *p* < 0.05)

Condition	NR	Glucose (mg/dl)	Triglyceride (mg/dL)	BUN (mg/dl)	Creatine (mg/dL)	ALT (U/L)	AST (U/L)	Corticosterone (ng/ml)	ACTH (ng/L)	Insulin (mIU/L)
N	0	113.86	75.62	23.06	0.41	99.29	114.14	56.69	38.68	17.71
	150	111.55	73.72	22.84	0.40	97.86	112.00	54.74	36.21	17.86
	300	108.97	74.50	21.91	0.40	96.43	110.57	53.38	37.07	17.13
	600	107.44	76.39	22.61	0.39	96.86	111.14	51.30	40.45	16.65
CVS	0	188.96	96.88	23.96	0.40	149.00	157.57	97.27	91.61	12.78
	150	171.63	88.27	22.71	0.39	136.00	151.43	79.17	80.37	13.84
	300	148.75	84.59	22.63	0.39	128.29	139.14	74.84	72.78	14.50
	600	139.37	81.62	22.41	0.39	124.14	130.29	65.69	67.39	15.04
*SEM*		4.05	1.36	0.01	0.23	2.74	1.33	2.12	2.89	0.31
*ANOVA*	Condition	0.001	0.001	0.487	0.511	0.001	0.001	0.001	0.001	0.001
	NR	0.001	0.410	0.267	0.909	0.001	0.001	0.001	0.001	0.656
	Condition x NR	0.001	0.033	0.767	0.994	0.001	0.008	0.001	0.001	0.026
*Polynomial Contrast*										
N	Linear	0.055	0.833	0.496	0.472	0.320	0.596	0.011	0.369	0.136
	Quadratic	0.875	0.565	0.876	0.515	0.633	0.757	0.964	0.065	0.581
	Cubic	0.903	0.914	0.967	0.456	0.831	0.948	0.838	0.905	0.664
CVS	Linear	0.001	0.001	0.754	0.093	0.001	0.001	0.001	0.001	0.007
	Quadratic	0.189	0.181	0.976	0.402	0.089	0.648	0.056	0.164	0.645
	Cubic	0.160	0.647	0.881	0.638	0.879	0.473	0.074	0.874	0.912

The data are presented as mean ± standard deviation. *BUN* blood urea nitrogen; *ALT* Alanine transaminase; *AST* Aspartate transaminase; *ACTH* Adrenocorticotropic hormone; *SEM* standard error of the mean

### Liver triglycerides and nicotinamide metabolites

Liver triglyceride levels increased in rats exposed to CVS compared to rats kept under normal conditions (*p* < 0.001, Table 3). In rats exposed to chronic variable stress factors, increasing doses of NR supplementation resulted in a linear decrease of approximately 18% in liver triglyceride levels (*p* < 0.001). Liver NAM levels were significantly lower in rats exposed to CVS compared to the normal group (*p* < 0.001, Table 3). With increasing doses of NR supplementation, a linear increase in NAM levels was observed, reaching approximately 49% in the normal group and 71% in the CVS group (*p* < 0.001, Table 3). Liver NAD + and NADPH levels showed a decrease in the CVS groups (*p* < 0.001, Table 3). In the normal group, a linear and quadratic increase of approximately 129% in NAD + levels and about 44% in NADPH levels was found with increasing doses of NR supplementation (*p* < 0.001). In the stress group, a linear increase of approximately 77% in NAD + levels and about 68% in NADPH levels was detected (*p* < 0.001). An interaction was detected between stress exposure and NR supplementation concerning NAD + and NADPH levels (*p* < 0.001 and *p* = 0.038, respectively). Liver NA concentration decreased with stress exposure (*p* < 0.001, Table 3). In the group not subjected to chronic variable stress, a linear increase of approximately 34% in NA concentration was observed with increasing doses of NR supplementation (*p* < 0.005). In the stress group, a linear increase of approximately 46% in NA concentration was found with increasing doses of NR supplementation (*p* < 0.001).

**Table 3:** The statistical analysis of the effect of different doses of nicotinamide riboside (NR) on biochemical parameters in normal (N) and chronic variable stress (CVS)-exposed rats (Two-way ANOVA and polynomial contrast, *p* < 0.05)

Condition	NR	Triglyceride (mg/g)	NAM (µmol/g)	NAD^+^ (µmol/g)	NADPH (nmol/g)	NA (µmol/g)
N	0	54.20	3.16	0.98	71.31	1.97
	150	53.60	3.71	1.31	86.28	2.26
	300	51.31	4.07	1.70	96.28	2.50
	600	48.92	4.71	2.25	103.06	2.64
CVS	0	74.43	1.69	0.45	35.23	1.21
	150	64.92	2.18	0.59	45.72	1.46
	300	62.60	2.66	0.70	52.80	1.60
	600	60.57	2.89	0.80	59.40	1.77
*SEM*		1.30	0.13	0.08	3.14	0.06
*ANOVA*	Condition	0.001	0.001	0.001	0.001	0.001
	NR	0.001	0.001	0.001	0.001	0.001
	Condition x NR	0.141	0.001	0.001	0.038	0.105
*Polynomial Contrast*						
N	Linear	0.086	0.001	0.001	0.001	0.001
	Quadratic	0.696	0.342	0.001	0.028	0.042
	Cubic	0.879	0.047	0.551	0.826	0.667
CVS	Linear	0,001	0.001	0.001	0.001	0.001
	Quadratic	0,109	0.001	0.293	0.069	0.190
	Cubic	0,500	0.094	0.838	0.528	0.248

The data are presented as mean ± standard deviation.* NAM* Nicotinamide; *NAD+* Nicotinamide adenine dinucleotide; *NADPH* Nicotinamide adenine dinucleotide phosphate; *NA* Nicotinic acid; *SEM* standard error of the mean

### Liver protein levels

**Fig. 3:**
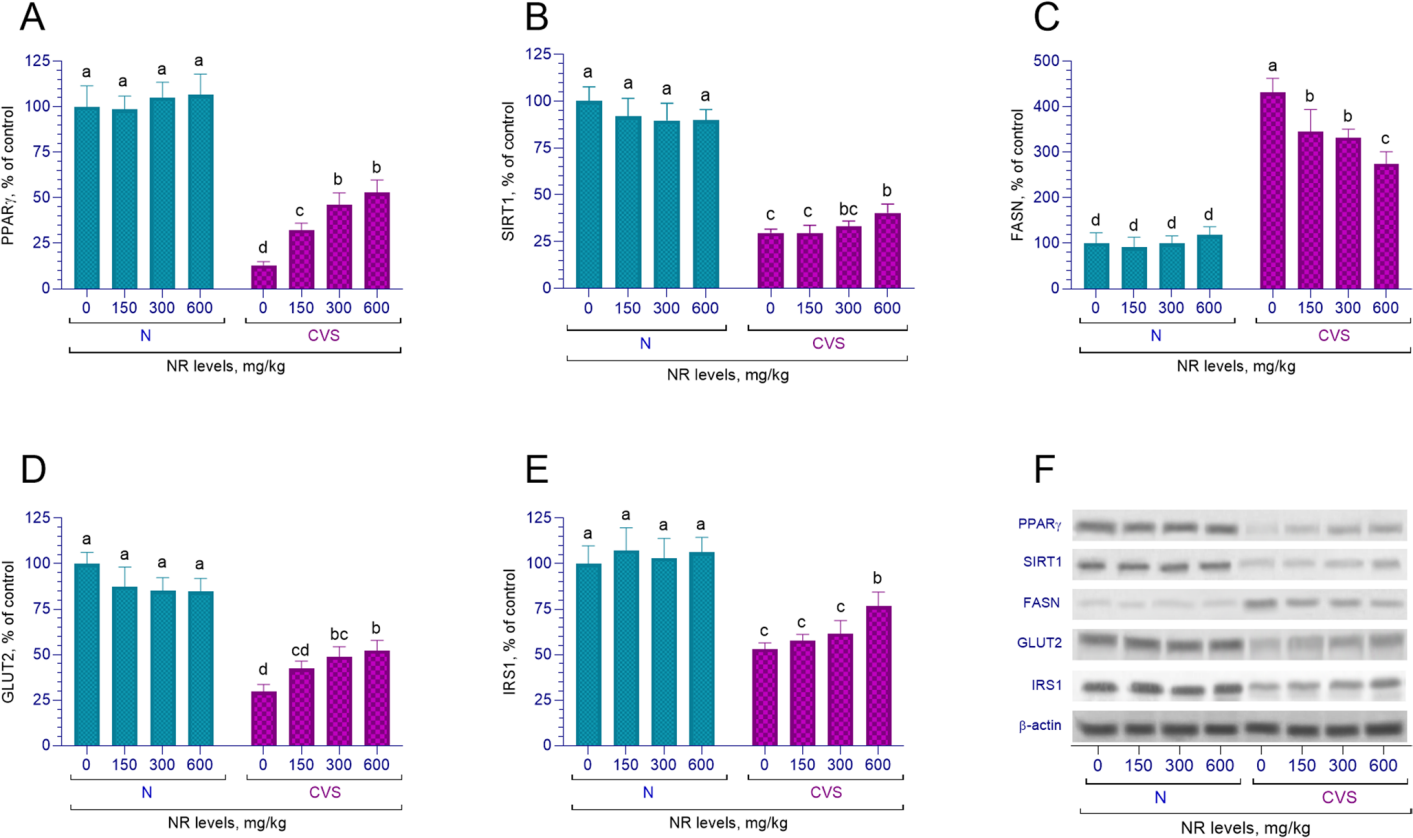
The effects of different nicotinamide riboside doses on the levels of PPARγ (**A**), SIRT1 (**B**), FASN (**C**), GLUT2 (**D**), and IRS1 (**E**) proteins in the liver tissue of rats under normal (N) and chronic variable stress (CVS) conditions. Protein levels were analyzed via Western blot using β-actin as a loading control to ensure equal protein loading. The representative bands are displayed in panel (**F**). Different letters (a–d) indicate statistically significant differences between groups (*p* < 0.05) according to Tukey’s post hoc test following two-way ANOVA. Two-way ANOVA revealed significant effects for PPARγ (Condition *p* < 0.0001; NR *p* < 0.0001; Condition x NR *p* < 0.0001), SIRT1 (Condition *p* < 0.0001; NR *p* = 0.1593; Condition x NR *p* = 0.0006), FASN (Condition *p* < 0.0001; NR *p* < 0.0001; Condition x NR *p* < 0.0001), GLUT2 (Condition *p* < 0.0001; NR *p* = 0.4003; Condition x NR *p* = 0.002), and IRS1 (Condition *p* < 0.0001; NR *p* = 0.0004; Condition x NR *p* = 0.0169). Comprehensive statistical data are presented in Supplementary Table A.2 and full immunoblots images are shown in supplementary Fig. A.1

The liver PPARγ protein levels were found to be decreased in animals exposed to chronic variable stress factors (*p* < 0.001). In the stress-exposed groups, NR supplementation dose-dependently increased PPARγ protein levels (*p* < 0.001, Fig. 3A). An interaction was observed between the stress condition and NR supplementation regarding PPARγ protein levels (*p* < 0.001). In the normal groups, NR supplementation did not cause any statistically significant change in PPARγ protein levels (*p* > 0.05). However, in the stress-exposed groups, a linear and quadratic increase in PPARγ protein levels was observed (*p* < 0.05). Stress exposure resulted in a decrease in SIRT1 protein levels (*p* < 0.001, Fig. 3B). It was determined that NR administered at different doses linearly increased SIRT1 protein levels in both normal and chronic variable stress-exposed groups (*p* < 0.001). FASN protein levels were found to be increased in stress-exposed rats compared to those not subjected to stress (*p* < 0.001, Fig. 3C). An interaction was identified between stress and NR supplementation concerning FASN levels (*p* < 0.001). In the normal groups, no significant effect of NR supplementation on FASN levels was observed (*p* > 0.05), whereas a linear decrease was noted in the groups exposed to stress (*p* < 0.001). The effect of stress led to a decrease in GLUT2 and IRS1 protein levels (*p* < 0.001, Fig. 3D and E). In the group subjected to chronic variable stress, NR supplementation resulted in an increase in both GLUT2 and IRS1 protein levels (*p* < 0.001). NR supplementation in the stressed group, a linear increase was observed (*p* < 0.001). Furthermore, in the chronic variable stress-exposed groups, linear and quadratic increases in IRS1 levels were noted (*p* < 0.05), while no significant effect on IRS1 levels was observed in the normal groups (*p* > 0.05).

### Jejunum protein levels

**Fig. 4:**
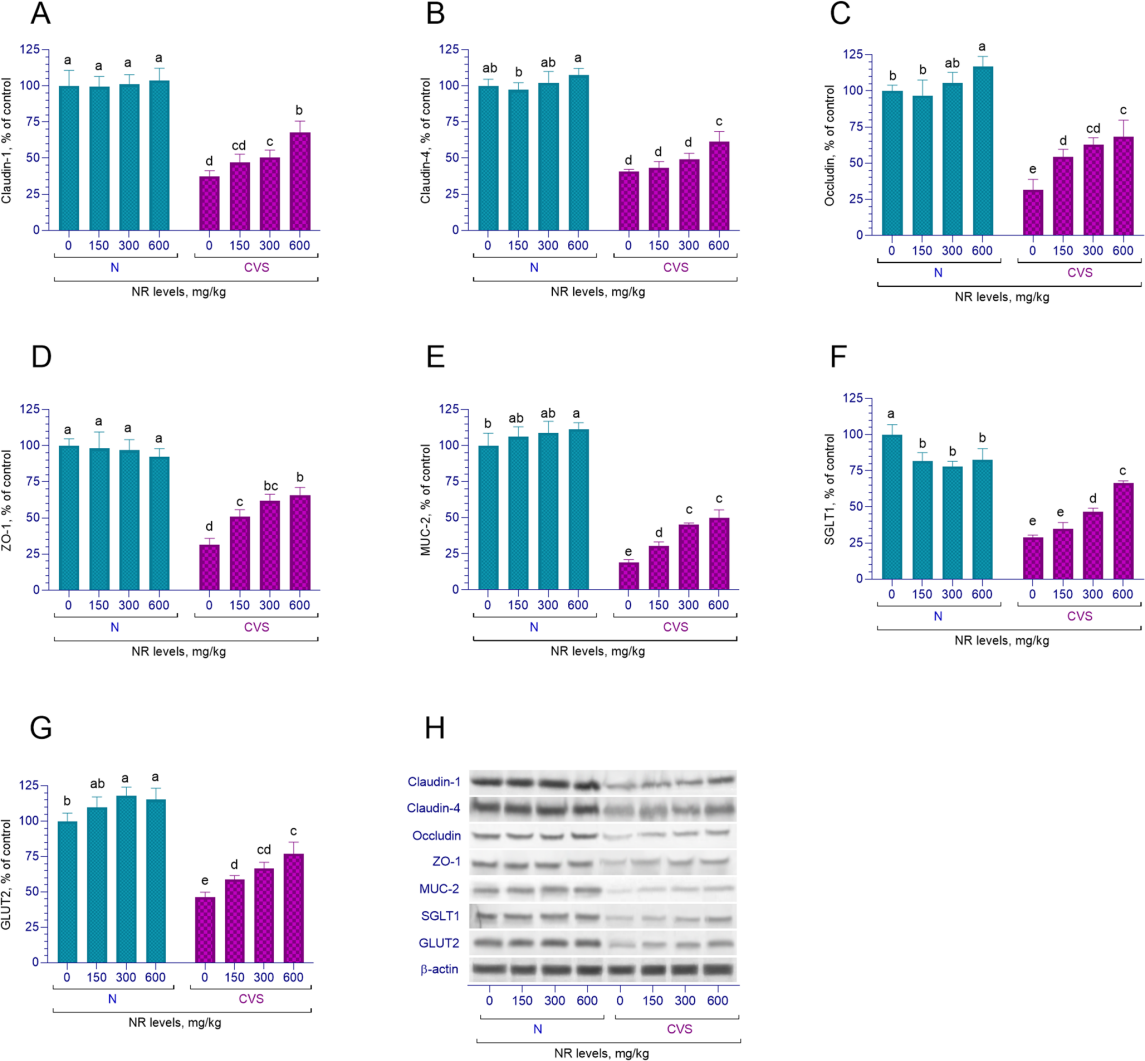
The effects of different nicotinamide riboside doses on the levels of Claudin-1 (**A**), Claudin-4 (**B**), Occludin (**C**), ZO-1 (**D**), MUC-2 (**E**), SGLT1 (**F**), and GLUT2 (**G**) proteins in the jejunum tissue of rats under normal (N) and chronic variable stress (CVS) conditions. Western blot analysis was performed using β-actin as a loading control to ensure equal protein loading. All bands are shown in (**H**). Different letters (a–e) indicate statistically significant differences between groups (*p* < 0.05) according to Tukey’s post hoc test following two-way ANOVA. Two-way ANOVA revealed significant effects for Claudin-1 (Condition *p* < 0.0001; NR *p* < 0.0001; Condition x NR *p* = 0.0002), Claudin-4 (Condition *p* < 0.0001; NR *p* < 0.0001; Condition x NR *p* = 0.0155), Occludin (Condition *p* < 0.0001; NR *p* < 0.0001; Condition x NR *p* < 0.0001), ZO-1 (Condition *p* < 0.0001; NR *p* < 0.0001; Condition x NR *p* < 0.0001), MUC-2 (Condition *p* < 0.0001; NR *p* < 0.0001; Condition x NR *p* < 0.0001), SGLT1 (Condition *p* < 0.0001; NR *p* < 0.0001; Condition x NR *p* = 0.0002), and GLUT2 (Condition *p* < 0.0001; NR *p* < 0.0001; Condition x NR *p* < 0.0001). Comprehensive statistical data are presented in Supplementary Table A.3 and full immunoblots images are shown in supplementary Fig. A.2

In this study, the levels of jejunal cell junction proteins claudin-1, ZO-1, and mucin protein MUC-2 were examined. It was found that in rats subjected to stress, the levels of these proteins decreased compared to the non-stressed group (*p* < 0.001, Fig. 4A, D and E). An interaction between stress conditions and NR supplementation was identified concerning the levels of claudin-1, ZO-1, and MUC-2 proteins (*p* < 0.001). In the normal group, a linear increase in MUC-2 levels was observed with increasing doses of NR supplementation (*p* < 0.001), while no significant differences were found in the levels of claudin-1 and ZO-1 proteins (*p* > 0.05). However, in the groups subjected to chronic variable stress, a linear increase in claudin-1 levels was observed, along with linear and quadratic increases in the levels of ZO-1 and MUC-2 proteins (*p* < 0.05). The level of jejunal claudin-4 protein was found to be decreased in stressed rats compared to the control group (*p* < 0.001, Fig. 4B). An interaction between stress conditions and NR supplementation regarding claudin-4 protein levels was noted (*p* < 0.05). A linear increase in claudin-4 protein levels was observed with increasing doses of NR supplementation in both stressed and non-stressed groups (*p* < 0.05). A decrease in the levels of jejunal occludin, SGLT1, and GLUT2 proteins was observed due to stress exposure (*p* < 0.001, Fig. 4C, F and G). There was an interaction between stress conditions and NR supplementation regarding the levels of occludin, SGLT1, and GLUT2 proteins (*p* < 0.05). In the groups subjected to stress, a linear and quadratic increase in occludin and GLUT2 protein levels was detected in parallel with the increasing doses of NR supplementation (*p* < 0.05), while a linear and quadratic decrease in SGLT1 protein levels was noted (*p* < 0.001). In the stressed group, a linear increase in occludin, SGLT1, and GLUT2 protein levels was observed with increasing doses of NR supplementation (*p* < 0.05).

**Fig. 5:**
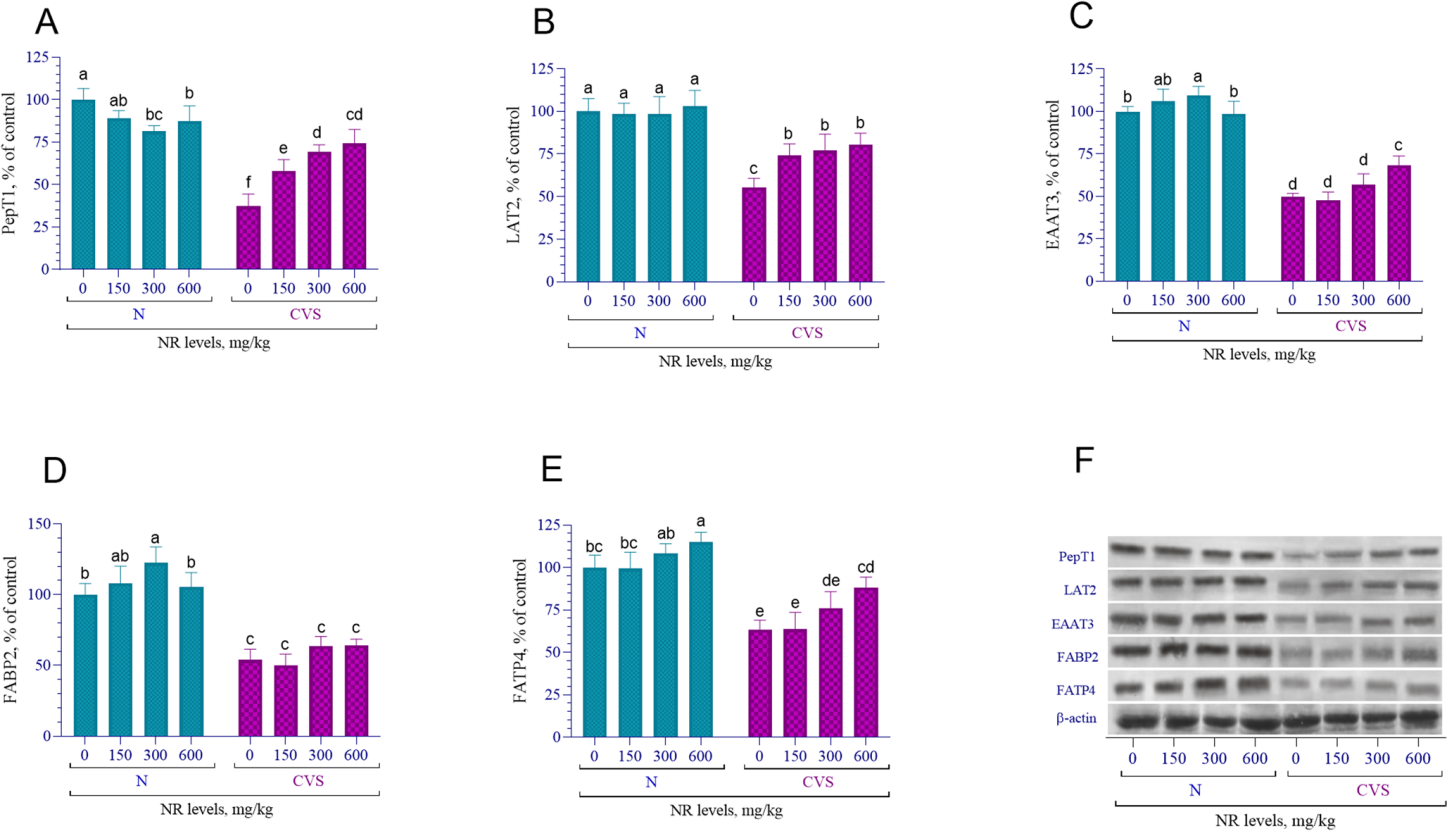
The effects of different nicotinamide riboside doses on the levels of PepT1 (**A**), LAT2 (**B**), EAAT3 (**C**), FABP2 (**D**), and FATP4 (**E**) proteins in the jejunum tissue of rats under normal and chronic variable stress (CVS) conditions. Western blot analysis was performed using β-actin as a loading control to ensure equal protein loading. All bands are shown in (G). Different letters (a–e) indicate statistically significant differences between groups (*p* < 0.05) according to Tukey’s post hoc test following two-way ANOVA. Two-way ANOVA revealed significant effects for PepT1 (Condition *p* < 0.0001; NR *p* = 0.0001; Condition x NR *p* < 0.0001), LAT2 (Condition *p* < 0.0001; NR *p* = 0.0002; Condition x NR *p* = 0.0007), EAAT3 (Condition *p* < 0.0001; NR *p* < 0.0001; Condition x NR *p* < 0.0001), FABP2 (Condition *p* < 0.0001; NR *p* < 0.0001; Condition x NR *p* = 0.0236), and FATP4 (Condition *p* < 0.0001; NR *p* < 0.0001; Condition x NR *p* = 0.3521). Comprehensive statistical data are presented in Supplementary Table A.4 and full immunoblots images are shown in supplementary Fig. A.3

In CVS-treated rats, protein levels of jejunum PepT1, LAT2, EAAT3, FABP2, and FATP4 were significantly reduced compared to the normal group (*p* < 0.0001, Fig. 5). NR supplementation at various doses led to a linear increase in PepT1, LAT2, and FATP4 protein levels in CVS groups (*p* < 0.001, Fig. 5A, B and C). Additionally, increases in PepT1 levels (linear, *p* < 0.001) and in FABP2 levels (linear *p* < 0.001) were identified in CVS-treated groups (Fig. 5A and D). Similarly, an increase in FATP4 levels was observed in both normal and CVS groups (linear, *p* < 0.001, Fig. 5E). An interaction between stress conditions and NR supplementation was observed for these proteins (*p* < 0.05).

### Histopathological analyses in the liver and jejunum

**Fig. 6:**
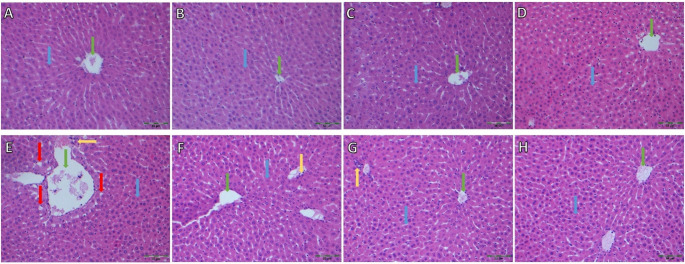
Histopathological appearance of hepatic tissue stained with Hematoxylin-Eosin (H&E): NR0 (**A**), NR150 (**B**), NR300 (**C**), NR600 (**D**), CVS-NR0 (**E**), CVS-NR150 (**F**), CVS-NR300 (**G**), CVS-NR600 (**H**). Blue arrow: hepatocytes, green arrow: central vein, yellow arrow: portal area, red arrow: fat vacuoles (H&E x200)

In the stress group, macro and microvesicular steatosis was observed in hepatocytes surrounding the central vein. No significant difference was found between the groups treated with NR and the control group (Fig. 6). The steatosis observed in the stress group was not present in the treatment groups.

**Fig. 7:**
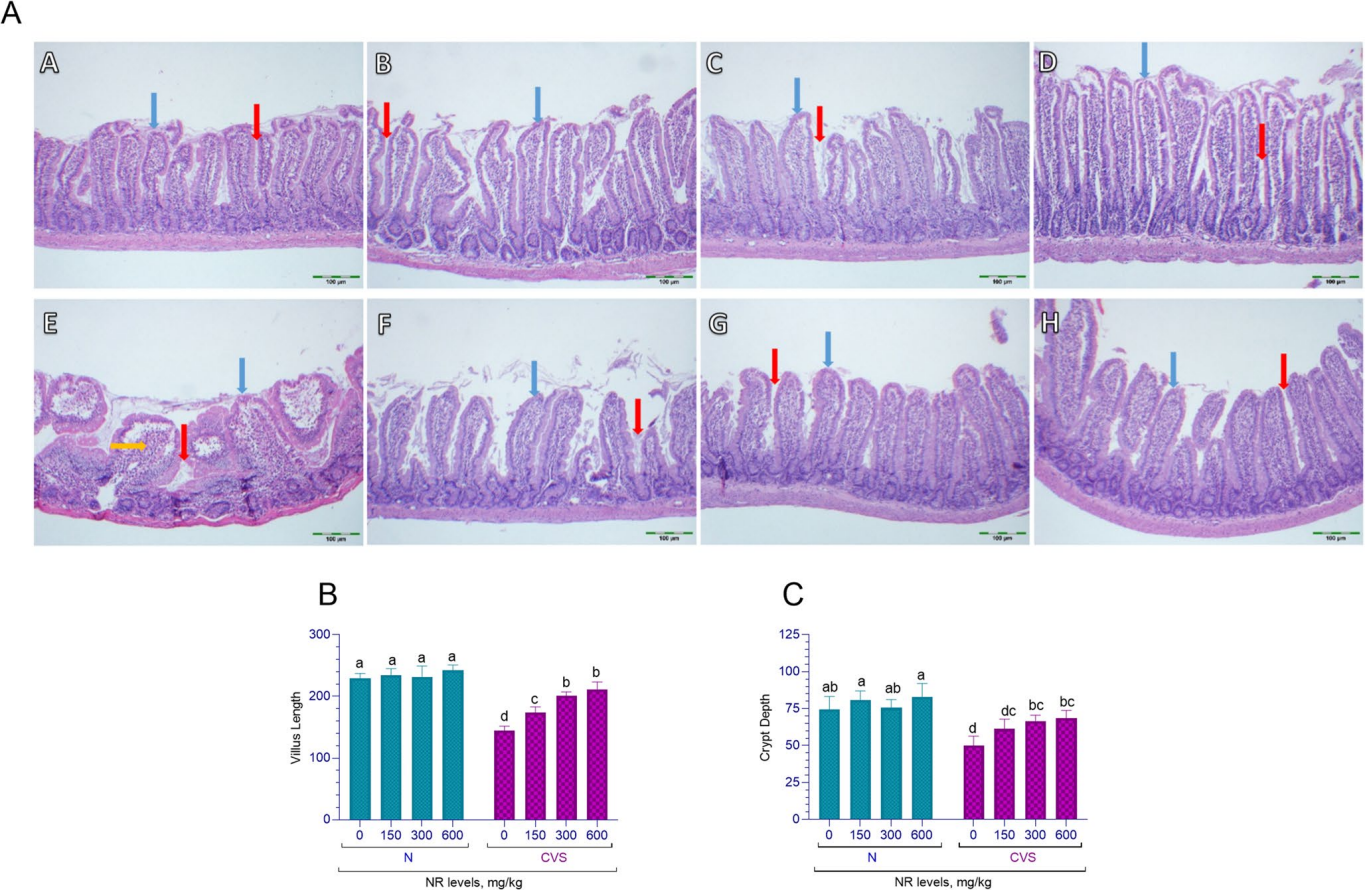
Histopathological appearance of jejunum tissue stained with Hematoxylin-Eosin (H&E): Panel **A**: NR0 (A), NR150 (B), NR300 (C), NR600 (D), CVS-NR0 (E), CVS-NR150 (F), CVS-NR300 (G), CVS-NR600 (H). (H&E x100). Blue arrow: villus; red arrow: crypt; yellow arrow: inflammation. Effects of different doses of nicotinamide riboside on villus length (Panel **B**) and crypt depth (Panel **C**) in jejunum tissue of normal (N) and chronic variable stress (CVS) exposed rats. Different letters (a–d) indicate statistically significant differences between groups (*p* < 0.05) according to Tukey’s post hoc test following two-way ANOVA. Two-way ANOVA revealed significant effects for villus length (Condition *p* < 0.0001; NR *p* = 0.0001; Condition x NR *p* < 0.0001), and crypt depth (Condition *p* < 0.0001; NR *p* < 0.0001; Condition x NR *p* = 0.0239). In addition, comprehensive statistical data can be found in Supplementary Table A.5

In rats subjected to chronic variable stress, it was observed that stress significantly reduced jejunum villus length (*p* = 0.003, Fig. 7B). An interaction between stress and nicotinamide riboside (NR) supplementation was identified (*p* = 0.003). In stressed rats, a linear and quadratic increase in villus length was noted with increasing doses of NR (*p* < 0.001 and *p* = 0.008), whereas no significant changes were detected in non-stressed rats (*p* > 0.05). Furthermore, stress exposure was found to decrease the crypt depth in the jejunum (*p* = 0.024, Fig. 7C). When examining the interaction between stress and NR on crypt depth, an interaction was also identified between these two factors (*p* < 0.001). In rats housed in normal conditions, NR supplementation did not show any effect on crypt depth (*p* > 0.05). However, in rats exposed to chronic variable stress, a linear and quadratic increase in crypt depth was observed with increasing doses of NR (*p* < 0.001 and *p* = 0.044). Comprehensive statistical data can be found in Supplementary Table S5.

Beyond microscopic evaluation, no clinical signs of toxicity such as reduced mobility, grooming abnormalities, or decreased food intake, were observed in any NR-only group during the study period. Body weight progression remained within physiological limits at all NR doses, and serum BUN and creatinine levels showed no indication of renal impairment.

## Discussion

This study was conducted to investigate the dose-dependent effects of NR supplementation on liver and intestinal health in rats exposed to chronic variable stress (CVS). Chronic stress is widely recognized for its detrimental impact on metabolic, endocrine, and structural homeostasis, particularly in the liver and intestine, where glucocorticoid excess induces oxidative stress, inflammation, and epithelial disruption [1, 3, 7]. Our findings demonstrate that NR exerts significant protective and restorative effects, supporting its potential use as a therapeutic agent in stress-induced metabolic disturbances.

Consistent with earlier studies, CVS markedly increased serum ALT and AST levels, indicating hepatocellular injury driven by glucocorticoid-mediated oxidative and inflammatory stress [50, 51]. NR supplementation dose-dependently normalized these enzymes, demonstrating strong hepatoprotection. Because NR has not previously been evaluated in a CVS model, dose selection was based on effective and well-tolerated ranges reported in neuropathy (200 mg/kg [41, 42]), metabolic dysfunction (400 mg/kg [23, 43]), and cardiometabolic disease models (100–400 mg/kg [40, 44]). Despite the highest dose (600 mg/kg) exceeding human clinical intake ranges, such differences reflect higher metabolic rate and NAD⁺ turnover in rodents. Across all evaluated endpoints including BUN, creatinine, ALT/AST, body weight, and histopathology, no NR-related toxicity was observed. However, we acknowledge that full toxicological characterization (organ weights, behavioral assessments, long-term exposure) is required for translational relevance.

Chronic stress has been shown to significantly increase serum corticosterone and ACTH levels, likely due to hyperactivation of the hypothalamic-pituitary-adrenal axis as part of the physiological stress response [51]. Elevated glucocorticoids are known to induce hyperglycemia by stimulating gluconeogenesis and glycogenolysis [52]. NR supplementation substantially reduced corticosterone, ACTH, and glucose concentrations in stressed rats, suggesting regulatory effects on neuroendocrine stress signaling and glucose homeostasis. CVS further disrupted lipid metabolism, as evidenced by increased triglyceride levels [53]. Whereas NR lowered triglycerides in a dose-dependent manner. Reduced insulin levels observed under CVS are in line with reports that chronic glucocorticoid elevation impairs insulin signaling [54]. NR restored insulin levels under stress, likely through SIRT1-dependent improvements in metabolic regulation [55].

Chronic stress is known to induce hepatic steatosis, reflected by increased hepatic triglyceride accumulation and histopathological damage [56]. Similarly, CVS increased hepatic triglycerides in our study, whereas NR supplementation significantly reduced these levels. Additionally, NR supplementation elevated hepatic NAM, NAD⁺, NADPH, and NA concentrations in both normal and CVS groups, consistent with research demonstrating that NR efficiently increases intracellular NAD⁺ pools [38]. Elevated NAD + levels are believed to play a critical role in preventing hepatic lipid accumulation, enhancing mitochondrial function, and mitigating the adverse effects of CVS on the liver by reducing oxidative stress [57]. In addition to NAD+, its precursors NA and NAM contribute to sustaining NAD + biosynthesis, which supports glucose homeostasis and reduces liver steatosis [58]. Furthermore, the observed increase in NADPH levels reinforces liver health by preventing oxidative damage, maintaining cellular redox balance, and reducing inflammation [59]. These findings collectively underscore the pivotal role of NR supplementation and its metabolites in protecting and supporting liver health, particularly under conditions of chronic stress.

At the molecular level, CVS induced marked reductions in PPARγ, SIRT1, SREBF1, FASN, GLUT2, and IRS1 protein levels, each of which plays a crucial role in regulating hepatic lipid and glucose metabolism. PPARγ is protective against liver fibrosis and metabolic dysfunction [60, 61]; its normalization by NR suggests mitigation of stress-related fibrogenic processes. CVS-induced suppression of SIRT1, previously associated with hepatic steatosis and inflammation [23, 62], was dose-dependently reversed by NR. Likewise, CVS-driven activation of the SREBF1-FASN lipogenic pathway [63] was suppressed by NR supplementation, demonstrating reversal of stress-induced lipogenesis. Decreased GLUT2 expression, a hallmark of impaired hepatic glucose handling, was alleviated by NR in agreement with Chen et al., 2020 [64]. The NR-mediated restoration of IRS1, a key regulator of insulin signaling and cell survival [65], further confirms NR’s role in improving metabolic function under stress.

The biomarker panel used in this study was intentionally selected to represent four mechanistically interconnected metabolic and epithelial pathways known to be disrupted by CVS, rather than redundant endpoints. GLUT2 and IRS1 were chosen as central markers of glucose–insulin homeostasis because glucocorticoid excess suppresses both glucose uptake and insulin signaling, and their normalization by NR reflects restoration of NAD⁺/SIRT1-dependent metabolic regulation [12, 66]. SREBF1 and FASN were included as indices of hepatic lipogenesis, as CVS activates the SREBF1-FASN axis and promotes steatosis [13, 40]. PepT1, LAT2, and EAAT3 were evaluated to capture amino acid and peptide absorption pathways, which influence insulin secretion, mTOR signaling, and epithelial metabolic adaptation, and are highly sensitive to stress-induced mucosal injury [15, 16]. FABP2 and FATP4 represented long-chain fatty acid uptake and intracellular trafficking, processes linked to intestinal barrier function, systemic inflammation, and insulin resistance [17, 18]. Integrating these markers provides a coherent mechanistic framework showing how CVS disrupts interconnected metabolic networks and how NR counteracts these alterations by restoring NAD⁺-dependent metabolic and epithelial pathways.

Chronic stress is well-documented to compromise intestinal barrier integrity and increase intestinal permeability, primarily by affecting tight junction proteins. Zheng et al. demonstrated that chronic stress reduces claudin-1 levels, a key tight junction protein in the intestinal epithelium, weakening the barrier function [67]. Similarly, Machorro-Rojas et al. found that chronic immobilization stress significantly lowers occludin and claudin-4 levels in the colon epithelium, leading to colon barrier dysfunction [68]. Additionally, stress-induced reductions in ZO-1 protein levels have been linked to exacerbating conditions such as inflammatory bowel disease [69].

The MUC2 protein, which forms a protective mucus layer over the intestinal epithelium and is vital for maintaining intestinal homeostasis, is also affected by chronic stress. Shigeshiro et al. reported that exposure to repeated water immersion stress reduces the number of goblet cells and suppresses the levels of MUC2 secreted by these cells, resulting in a decrease in colon MUC2 levels [10]. In the present study, similar significant decreases in claudin-1, claudin-4, occludin, ZO-1, and MUC2 levels were observed in the CVS groups. Notably, NR supplementation reversed these reductions, improving the levels of these proteins. This suggests that NR supplementation helps maintain intestinal barrier integrity, reduces permeability to pathogens, and supports glucose absorption. These results highlight the beneficial effects of NR on gut health. There are inconsistencies in the literature regarding SGLT1 levels under stress, with Chooi Yeng et al. reporting no changes in GLUT2 levels but observing an increase in SGLT1 levels in the jejunum and ileum [70]. In contrast, our study found decreases in both GLUT2 and SGLT1 levels in the CVS groups. These differences suggest that stress may have variable effects on glucose transport proteins, highlighting the need for further research to understand these mechanisms better.

There is limited information in the literature on the effects of chronic variable stress (CVS) on the protein levels of PepT1, LAT2, EAAT3, FABP2, and FATP4 in the jejunum. Therefore, direct comparison of the nutrient transporter data from this study with existing findings is not possible. However, previous studies suggest that stress increases intestinal permeability and can negatively impact the function of transporter proteins [71, 72]. In this study, CVS was found to reduce the protein levels of PepT1, LAT2, EAAT3, FABP2, and FATP4 in rats compared to the control group. Notably, NR treatment improved the levels of these proteins in CVS-exposed rats. Additionally, chronic stress has been shown to cause structural changes in the gut, such as villus atrophy and reduced crypt depth [7]. Our findings align with these observations, as we also noted similar atrophic changes in stressed groups. However, NR treatment demonstrated significant protective effects, reversing villus atrophy and increasing crypt depth. These results suggest that NR supplementation has a restorative and protective effect on gastrointestinal alterations caused by chronic stress.

Emerging evidence further contextualizes our findings by showing that NR may exert context-dependent or even bidirectional effects in stress environments. In aged mice, NR increased stress sensitivity and altered hematopoietic homeostasis under chronic stress conditions [32]. Similarly, in chronic corticosterone exposure, NR modulated mitochondrial function, oxidative-inflammatory signaling, and behavioral stress vulnerability [33]. These studies indicate that NR’s biological effects depend on the physiological environment and degree of glucocorticoid burden. Given that CVS induces persistent glucocorticoid excess, mitochondrial redox imbalance, tight junction destabilization, mucin depletion, epithelial atrophy, and metabolic suppression, the CVS paradigm is a robust model for evaluating NR’s actions. The present findings demonstrate that NR supplementation restores NAD⁺ bioavailability, supports SIRT1-dependent mitochondrial and metabolic activity, normalizes lipid and glucose regulatory pathways, and preserves intestinal epithelial integrity.

The beneficial effects of NR observed in this study are attributable to its ability to replenish NAD⁺ pools essential for cellular energy production, DNA repair, and metabolic regulation. By enhancing NAD⁺-dependent processes, including SIRT1 activation, NR effectively reduces oxidative stress, inflammation, and lipid accumulation, thereby supporting coordinated liver–gut axis health under chronic stress conditions.

## Conclusions

In conclusion, the present study has shown that the application of NR supplementation, particularly at a high dose (600 mg/kg), resulted in an increase in liver NAD + and metabolite levels in rats exposed to CVS; furthermore, it demonstrated an improvement in the levels of liver metabolic regulatory proteins, potentially alleviating metabolic abnormalities. Additionally, the increase in adhesion and tight junction proteins in the intestine, which had decreased due to stress as a result of NR supplementation, could improve intestinal barrier integrity. NR supplementation has been found to alleviate the adverse effects of stress on liver and gut health. Future studies should explore the translational potential of NR in clinical settings and its broader applicability across other stress-related health conditions.

## Supplementary Information

Below is the link to the electronic supplementary material.

Supplementary Material 1

## Data Availability

The datasets used and analyzed in this current study are available from the corresponding author upon reasonable request.
