# Supplementary material for: Modulation of SIRT1/PPARγ pathways and tight junction proteins by nicotinamide riboside under chronic variable stress
Source: J Physiol Biochem. 2026 Jan 22;82(1):3. doi: 10.1007/s13105-026-01153-7 (PMC12823639; doi:10.1007/s13105-026-01153-7)
Supplement: Supplementary file 1 — Supplementary Material 1 [file 13105_2026_1153_MOESM1_ESM.docx]

**Supplementary tables and figures**

**Modulation of SIRT1/PPARγ Pathways and Tight Junction Proteins by Nicotinamide Riboside under Chronic Variable Stress**

**Abdullah Celik^1^, Nurhan Sahin^1^, Cemal Orhan^1^, Besir Er^2^, Fusun Erten^3^, Busra Ozmen^1^, Mehmet Tuzcu^4^, Ibrahim Hanifi Ozercan^5^, Kazim Sahin^1,*^**

^1^Department of Animal Nutrition, Faculty of Veterinary Medicine, Firat University, 23119, Elazig, Türkiye

^2^Department of Medical Services and Techniques, Vocational School of Health Services, Firat University, 23119, Elazig, Turkiye

^3^Department of Veterinary Science, Pertek Vocational School, Munzur University, 62500, Tunceli, Türkiye

^4^Department of Biology, Faculty of Science, Firat University, 23119, Elazig, Türkiye

^5^Department of Pathology, Medicine Faculty, Health Sciences Institution, Firat University, 23119, Elazig, Türkiye

***Correspondence:** Kazim Sahin, DVM, Ph.D., Professor of Nutrition, Veterinary Faculty, Firat University, 23119 Elazig, Türkiye. Phone: +90 424 237 00 00 ext: 3938, Fax: +90 424 238 81 73, Email: nsahinkm@yahoo.com

**Table A.1** Statistical analysis of final body weight with nicotinamide riboside (NR) supplementation in normal (N) and chronic variable stress (CVS) exposed rats. (Statistical Analysis Two-way ANOVA and Polynomial Contrast, p < 0.05)

| **ANOVA *P*** | | |
| --- | --- | --- |
| Condition | | 0.001 |
| NR | | 0.045 |
| Condition x NR | | 0.970 |
| *Polynomial Contrast* | | |
| N | Linear | 0.027 |
|  | Quadratic | 0.828 |
|  | Cubic | 0.967 |
| CVS | Linear | 0.082 |
|  | Quadratic | 0.809 |
|  | Cubic | 0.779 |
| NR: Nicotinamide Riboside, N: Normal Group, CVS: Chronic Variable Stress Group | | |

**Table A.2** The effect of NR on liver protein levels in groups exposed to normal and chronic variable stress. (Statistical Analysis Two-way ANOVA and Polynomial Contrast, p < 0.05)

|  |  | **PPARy** | **SIRT1** | **FASN** | **GLUT2** | **IRS1** |
| --- | --- | --- | --- | --- | --- | --- |
| *ANOVA* | Condition | 0.001 | 0.001 | 0.001 | 0.001 | 0.001 |
|  | NR | 0.001 | 0.159 | 0.001 | 0.400 | 0.001 |
|  | Condition x NR | 0.001 | 0.001 | 0.001 | 0.002 | 0.017 |
| *Polynomial Contrast* | | | | | | |
| N | Linear | 0.129 | 0.025 | 0.080 | 0.002 | 0.413 |
|  | Quadratic | 0.716 | 0.202 | 0.086 | 0.057 | 0.619 |
|  | Cubic | 0.427 | 0.810 | 0.875 | 0.524 | 0.298 |
| CVS | Linear | 0.001 | 0.001 | 0.001 | 0.001 | 0.001 |
|  | Quadratic | 0.004 | 0.012 | 0.283 | 0.020 | 0.029 |
|  | Cubic | 0.835 | 0.955 | 0.048 | 0.739 | 0.221 |
| N: Normal group; CVS: Chronic variable stress group; NR: Nicotinamide riboside; PPARγ: Peroxisome proliferator-activated receptor gamma; SIRT1: Sirtuin 1; FASN: Fatty acid synthase; GLUT2: Glucose transporter-2; IRS1: Insulin receptor substrate-1 | | | | | | |

**Table A.3** The effect of nicotinamide riboside (NR) on jejunum protein levels in groups exposed to normal (N) and chronic variable stress (CVS). (Statistical Analysis Two-way ANOVA and Polynomial Contrast, p < 0.05)

|  |  | **Claudin-1** | **Claudin-4** | **Occludin** | **ZO-1** | **MUC2** | **SGLT1** | **GLUT2** |
| --- | --- | --- | --- | --- | --- | --- | --- | --- |
| *ANOVA* | Condition | 0.001 | 0.001 | 0.001 | 0.001 | 0.001 | 0.001 | 0.001 |
|  | NR | 0.001 | 0.001 | 0.001 | 0.001 | 0.001 | 0.001 | 0.001 |
|  | Condition x NR | 0.001 | 0.015 | 0.001 | 0.001 | 0.001 | 0.001 | 0.007 |
| *Polynomial Contrast* | | | | | | | | |
| N | Linear | 0.376 | 0.009 | 0.001 | 0.078 | 0.005 | 0.001 | 0.001 |
|  | Quadratic | 0.608 | 0.081 | 0.020 | 0.655 | 0.521 | 0.001 | 0.023 |
|  | Cubic | 0.933 | 0.455 | 0.494 | 0.751 | 0.805 | 0.590 | 0.422 |
| CVS | Linear | 0.001 | 0.001 | 0.001 | 0.001 | 0.001 | 0.001 | 0.001 |
|  | Quadratic | 0.080 | 0.013 | 0.006 | 0.001 | 0.008 | 0.001 | 0.559 |
|  | Cubic | 0.058 | 0.761 | 0.373 | 0.823 | 0.019 | 0.595 | 0.411 |
| N: Normal group; CVS: Chronic variable stress group; NR: Nicotinamide riboside; ZO-1: Zona occludens-1; MUC-2: Mucin-2; SGLT1: Sodium-Glucose cotransporter-1; GLUT2: Glucose transporter-2 | | | | | | | | |

**Table A.4** The effect of nicotinamide riboside (NR) on jejunum protein levels in groups exposed to normal and chronic variable stress (CVS). (Statistical Analysis Two-way ANOVA and Polynomial Contrast, p < 0.05)

|  |  | **PepT1** | **LAT2** | **EAAT3** | **FABP2** | **FATP4** |
| --- | --- | --- | --- | --- | --- | --- |
| *ANOVA* | Condition | 0.001 | 0.001 | 0.001 | 0.001 | 0.001 |
|  | NR | 0.001 | 0.001 | 0.001 | 0.001 | 0.001 |
|  | Condition x NR | 0.001 | 0.001 | 0.001 | 0.024 | 0.352 |
| *Polynomial Contrast* | | | | | | |
| N | Linear | 0.001 | 0.515 | 0.001 | 0.086 | 0.001 |
|  | Quadratic | 0.001 | 0.359 | 0.001 | 0.004 | 0.186 |
|  | Cubic | 0.303 | 0.836 | 0.001 | 0.045 | 0.337 |
| CVS | Linear | 0.001 | 0.001 | 0.821 | 0.001 | 0.001 |
|  | Quadratic | 0.006 | 0.012 | 0.001 | 0.394 | 0.063 |
|  | Cubic | 0.758 | 0.203 | 0.362 | 0.014 | 0.375 |

N: Normal group; CVS: Chronic variable stress group; NR: Nicotinamide riboside, **PepT1:** Peptide transporter 1, LAT2: L-type amino acid transporter 2, EAAT3: Excitatory amino acid transporter 3, FABP2: Fatty acid binding protein 2, FATP4: Fatty acid transport protein 4.

**Table A.5** Effects of different nicotinamide riboside doses on villus length (A) and crypt depth (B) in jejunum tissue of rats exposed to normal and chronic variable stress. (Statistical Analysis Two-way ANOVA and Polynomial Contrast, p < 0.05)

|  |  | **Villus Length (µm)** | **Crypt Depth (µm)** |
| --- | --- | --- | --- |
| *ANOVA* | Condition | 0.001 | 0.024 |
|  | NR | 0.001 | 0.001 |
|  | Condition x NR | 0.001 | 0.001 |
| *Polynomial Contrast* | | | |
| N | Linear | 0.078 | 0.131 |
|  | Quadratic | 0.512 | 0.873 |
|  | Cubic | 0.288 | 0.073 |
| CVS | Linear | 0.001 | 0.001 |
|  | Quadratic | 0.008 | 0.044 |
|  | Cubic | 0.342 | 0.799 |

N: Normal group; CVS: Chronic variable stress group; NR: Nicotinamide riboside


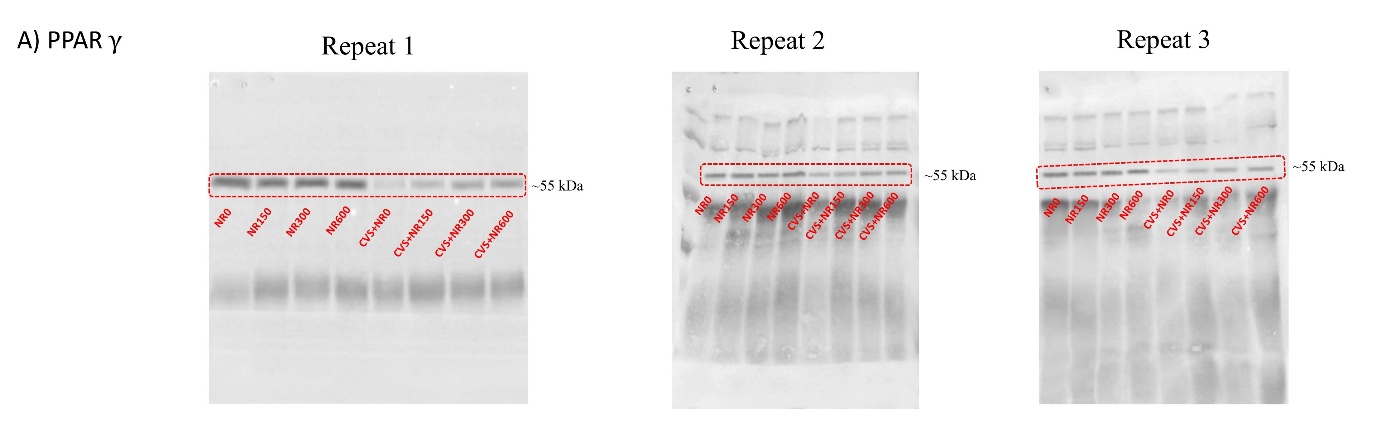

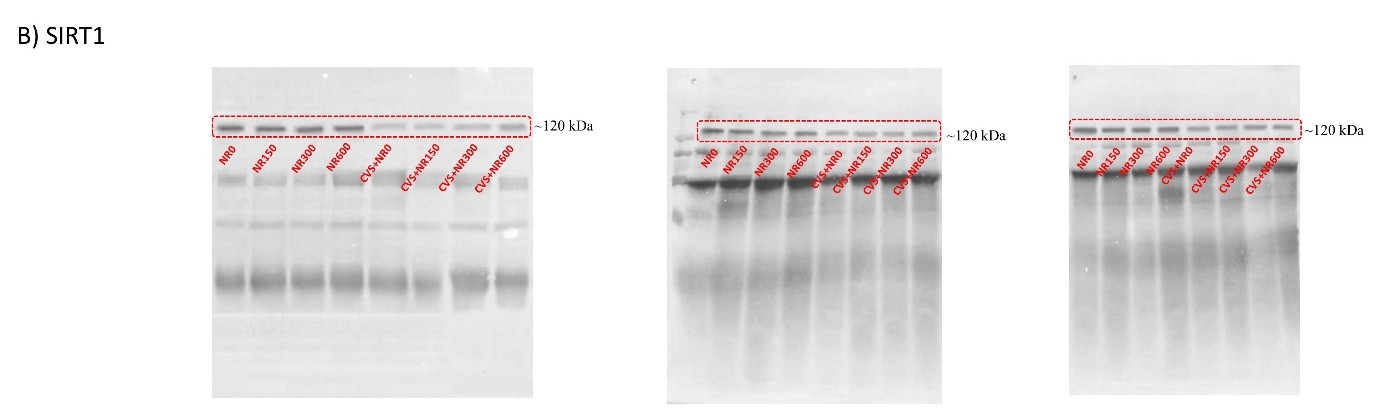

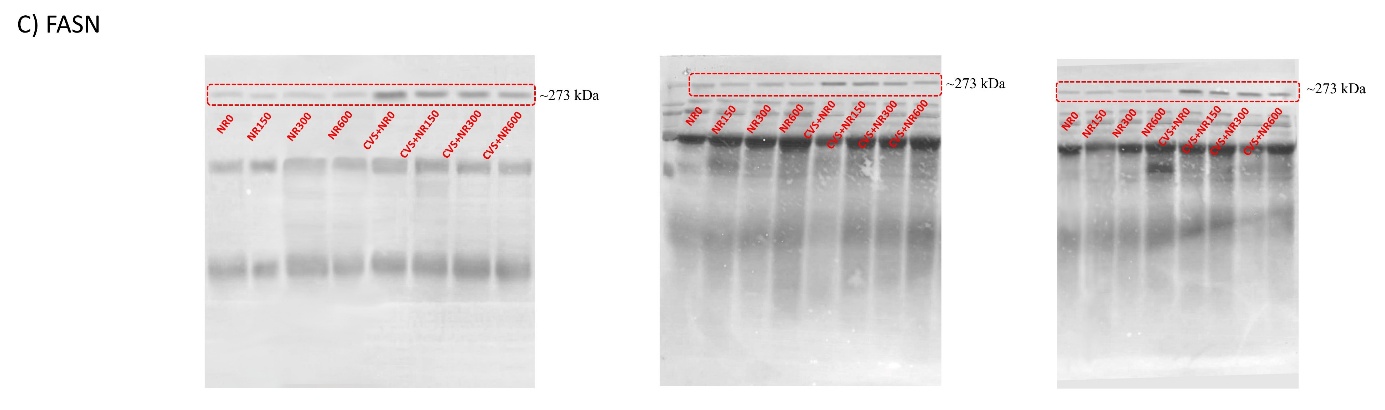

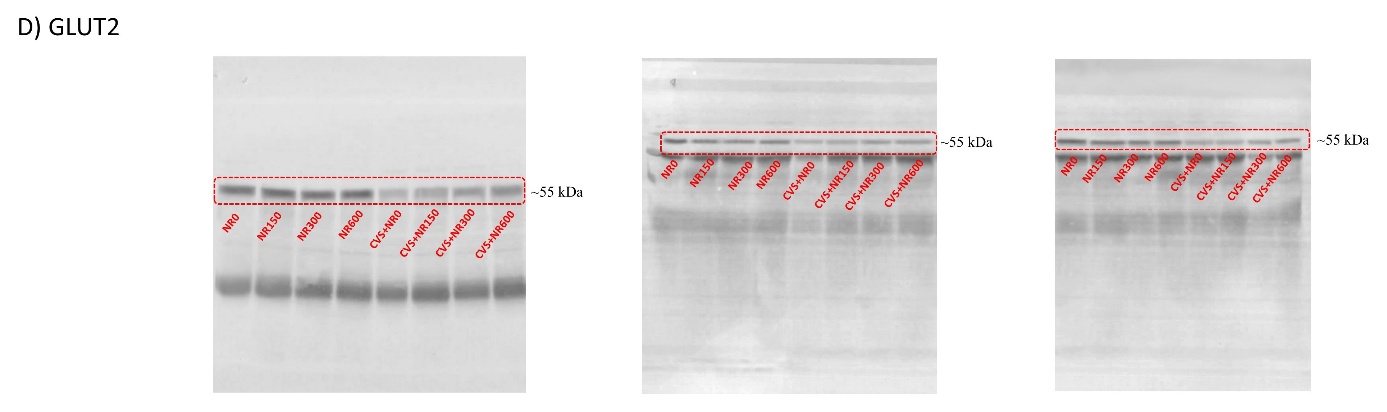

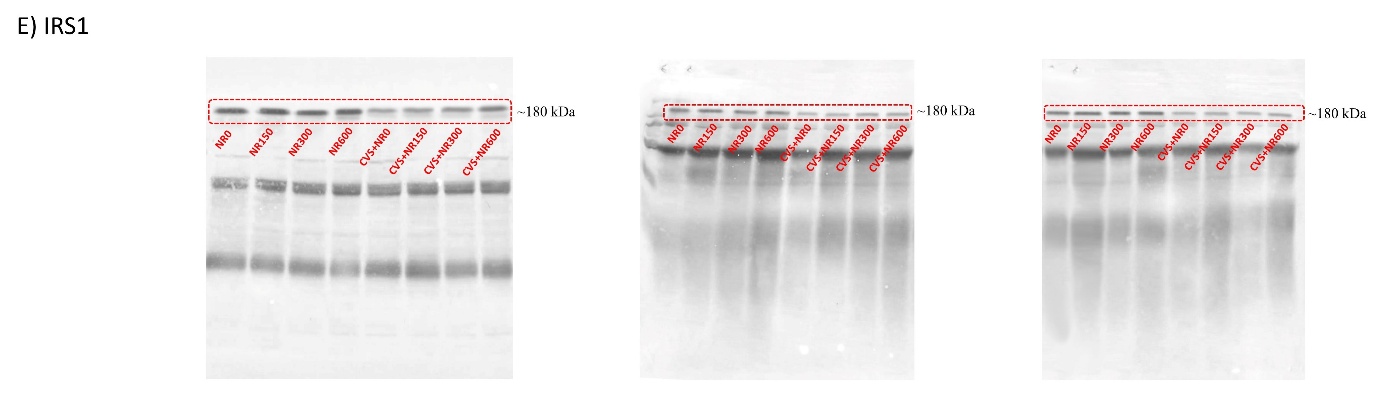

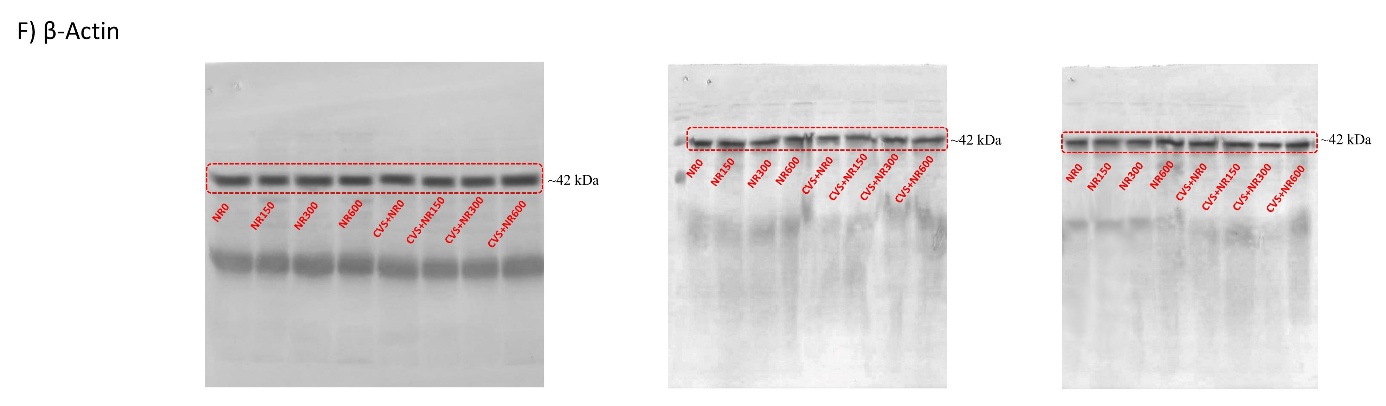


**Fig. A.1** Full immunoblots related to Fig. 3 in the main text: The effects of different nicotinamide riboside doses on the levels of PPARγ (A), SIRT1 (B), FASN (C), GLUT2 (D), and IRS1 (E) proteins in the liver tissue of rats under normal (N) and chronic variable stress (CVS) conditions. The densitometric analysis of the relative intensity according to the control group of the Western blotting bands was performed with β-actin normalization to ensure equal protein loading (F). All experiments were independently repeated three times. Red dotted rectangles delineate the results shown in Fig. 3 in the main text. Molecular weight (in kDa) is indicated. PPARγ: Peroxisome proliferator-activated receptor gamma; SIRT1: Sirtuin 1; FASN: Fatty acid synthase; GLUT2: Glucose transporter-2; IRS1: Insulin receptor substrate-1

**
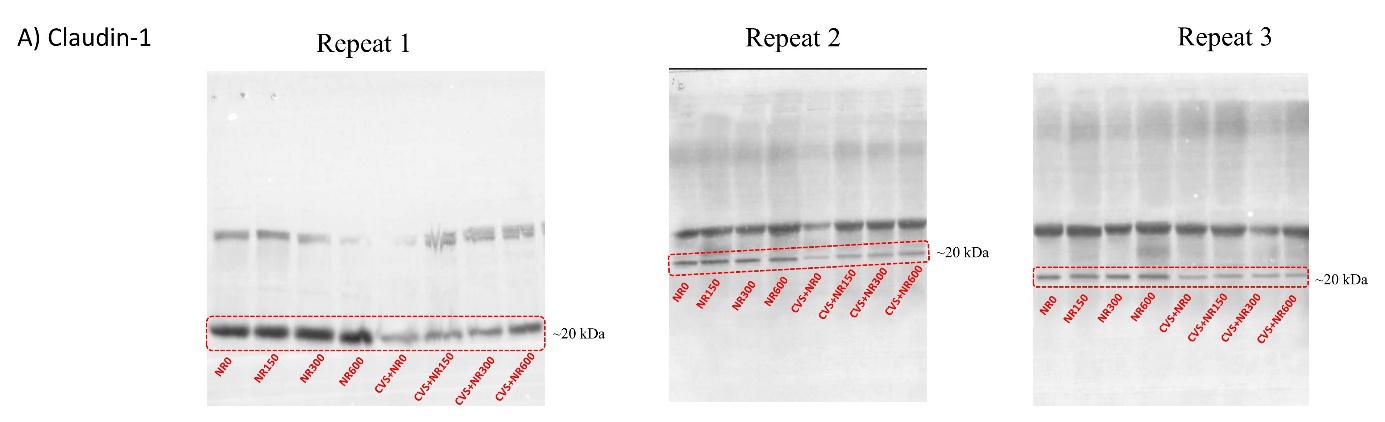

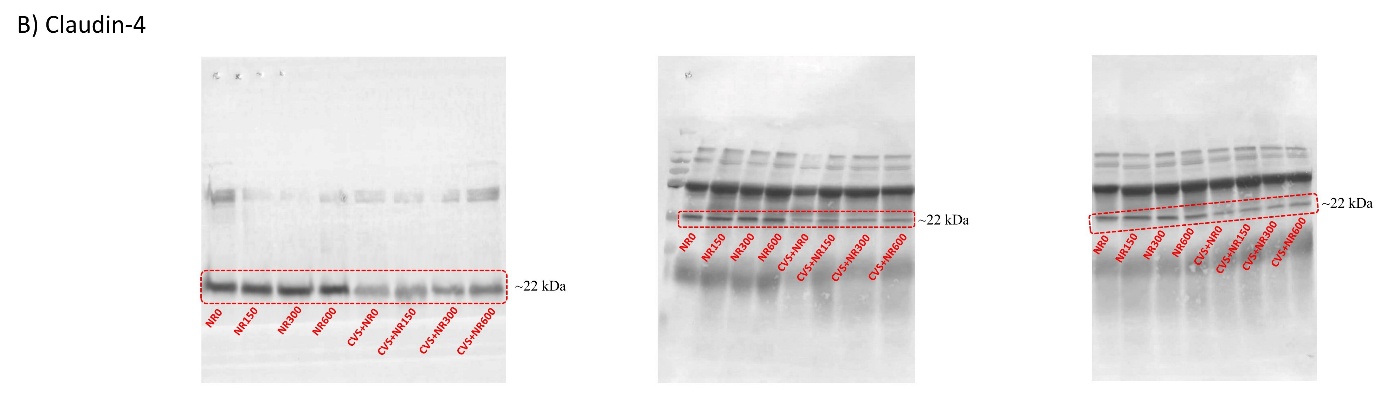

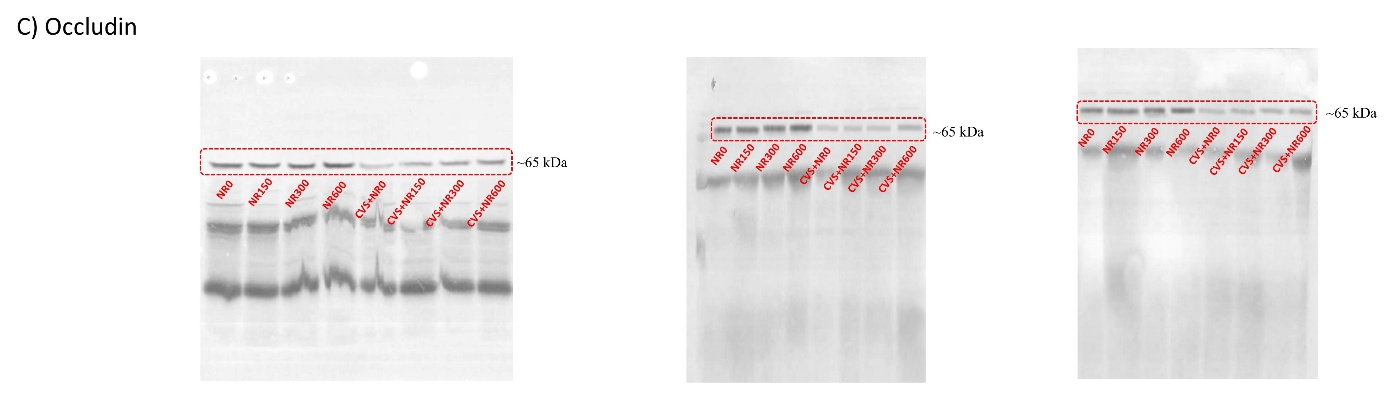

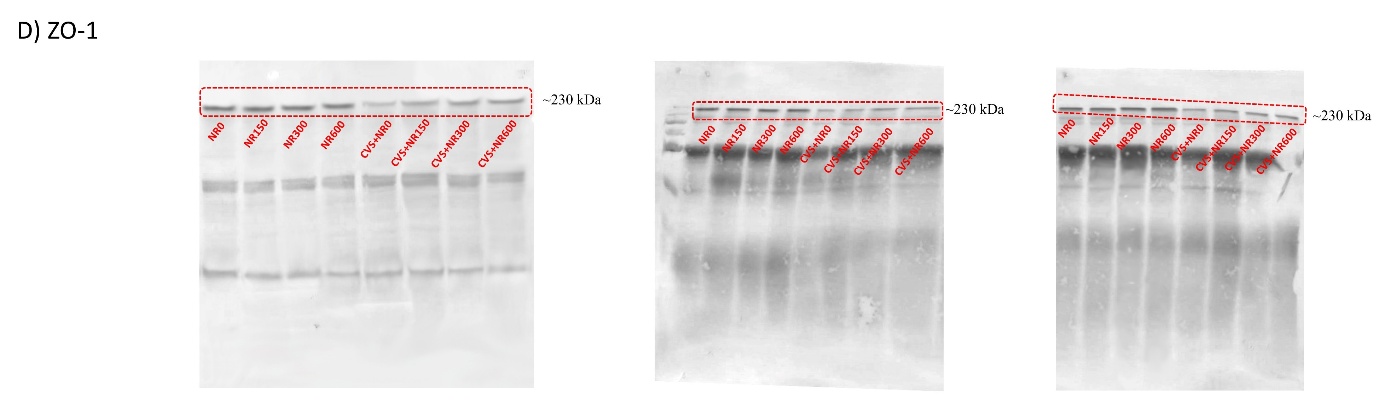

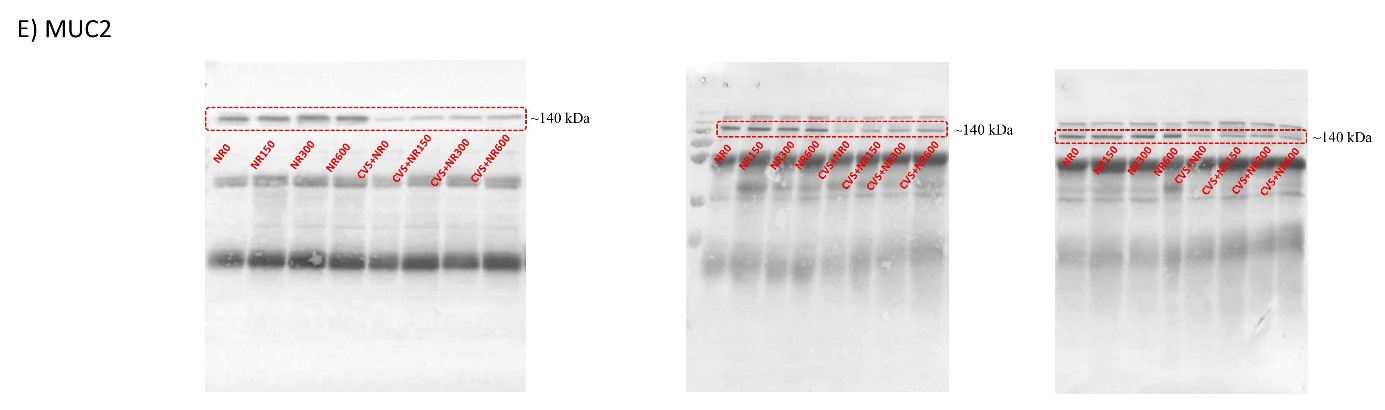

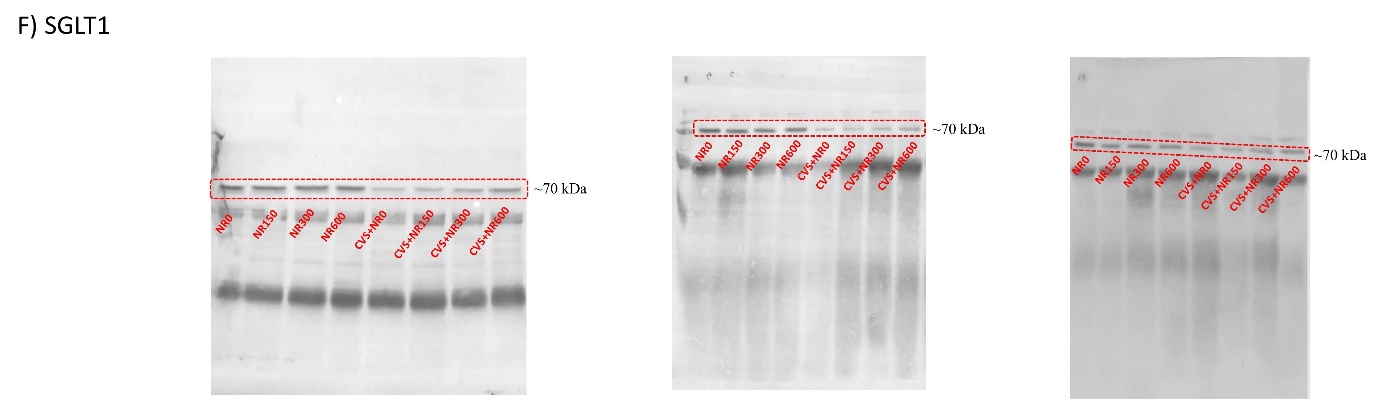

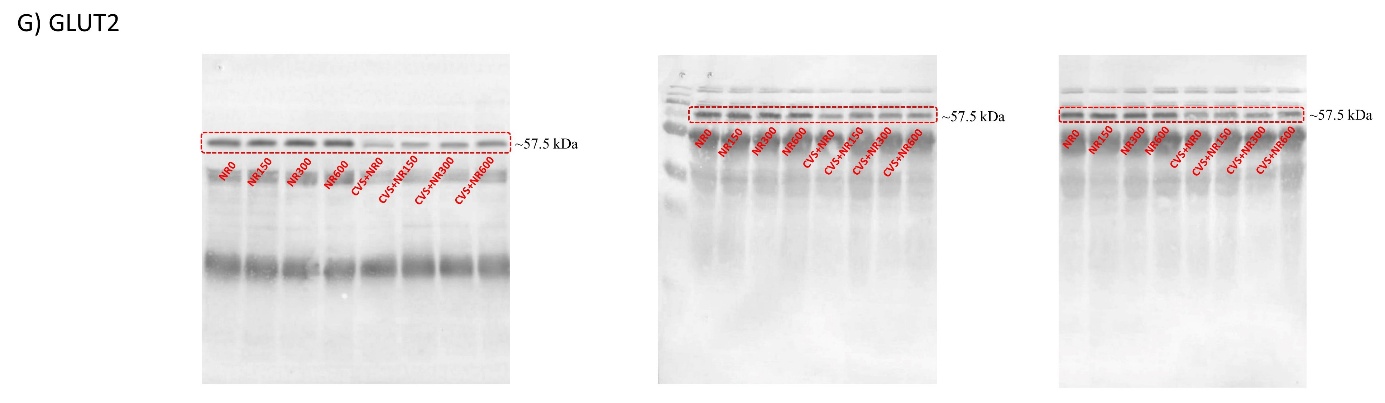
**


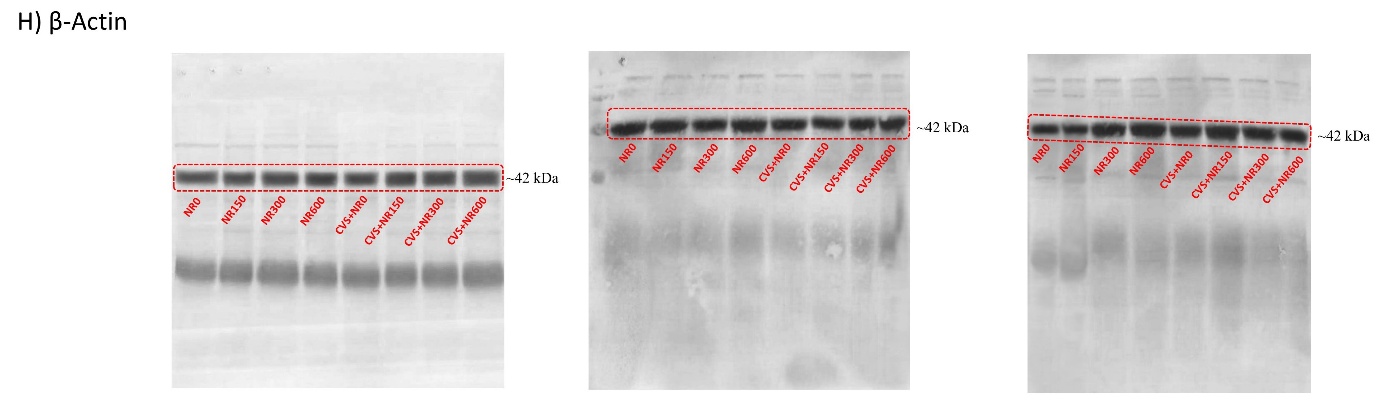


**Fig. A.2** Full immunoblots related to Fig. 4 in the main text: The effects of different nicotinamide riboside doses on the levels of Claudin-1 (A), Claudin-4 (B), Occludin (C), ZO-1 (D), MUC-2 (E), SGLT1 (F), and GLUT2 (G) proteins in the jejunum tissue of rats under normal (N) and chronic variable stress (CVS) conditions. The densitometric analysis of the relative intensity according to the control group of the Western blotting bands was performed with β-actin normalization to ensure equal protein loading (H). All experiments were independently repeated three times. Red dotted rectangles delineate the results shown in Fig. 4 in the main text. Molecular weight (in kDa) is indicated. ZO-1: Zona occludens-1; MUC-2: Mucin-2; SGLT1: Sodium-Glucose cotransporter-1; GLUT2: Glucose transporter-2

**
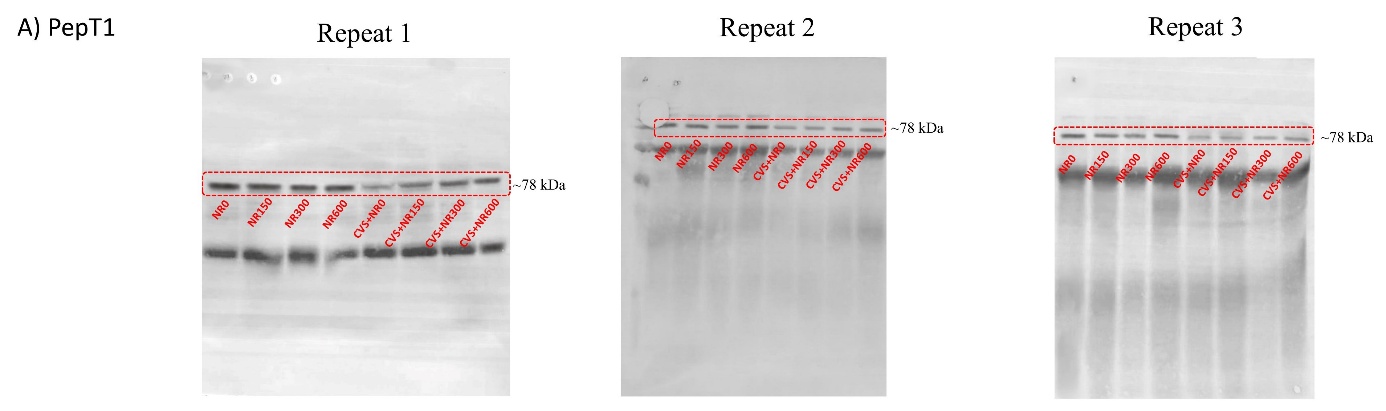

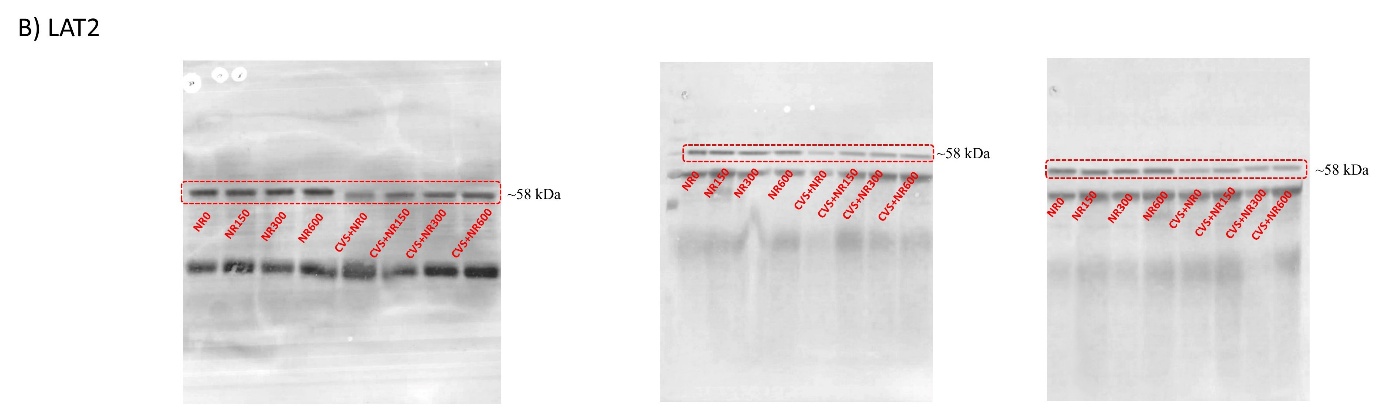

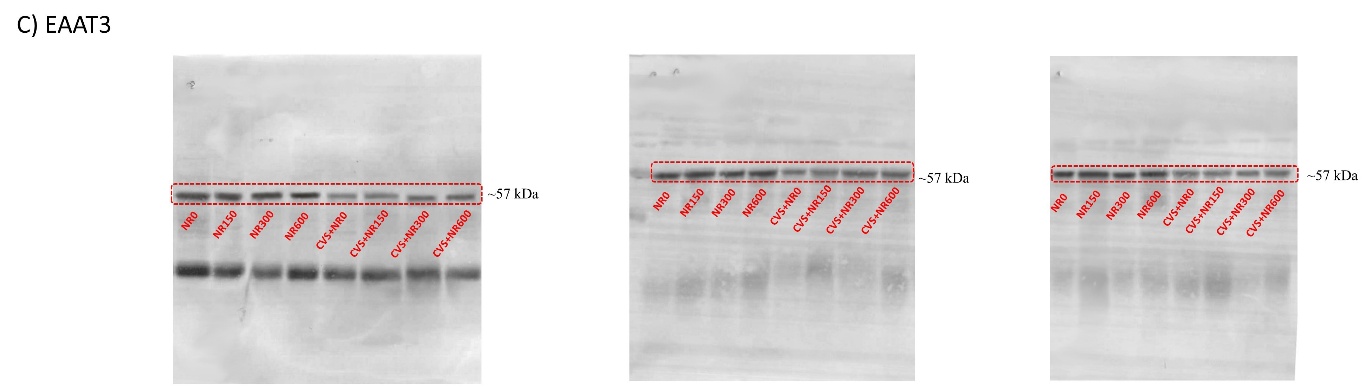

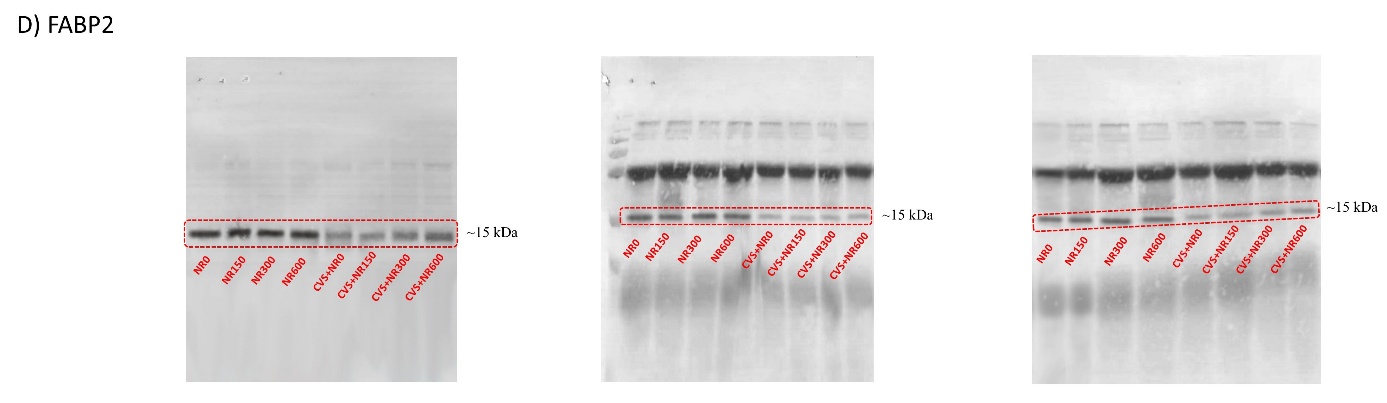

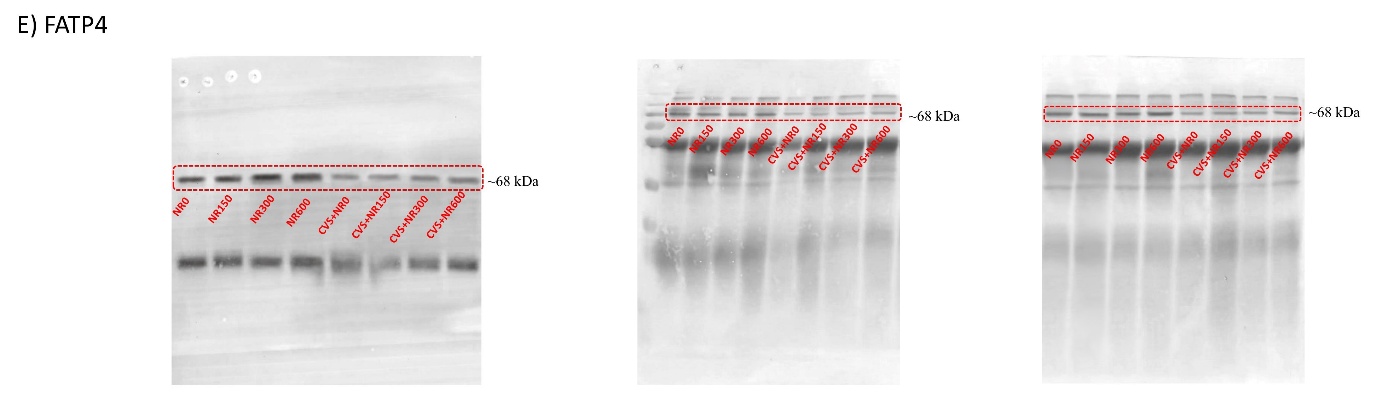

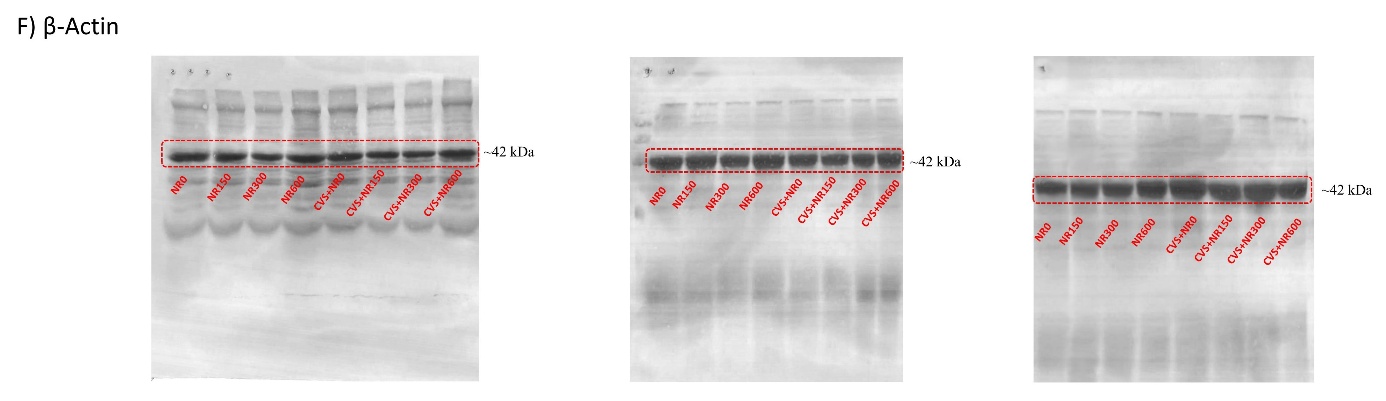
**

**Fig. A.3** Full immunoblots related to Fig. 5 in the main text: The effects of different nicotinamide riboside doses on the levels of PepT1 (A), LAT2 (B), EAAT3 (C), FABP2 (D), and FATP4 (E) proteins in the jejunum tissue of rats under normal (N) and chronic variable stress (CVS) conditions. The densitometric analysis of the relative intensity according to the control group of the Western blotting bands was performed with β-actin normalization to ensure equal protein loading (F). All experiments were independently repeated three times. Red dotted rectangles delineate the results shown in Fig. 5 in the main text. Molecular weight (in kDa) is indicated. PepT1, Peptide transporter 1; LAT2, L-Type amino acid transporter 2; EAAT3, Excitatory amino acid transporter 3; FABP2, Fatty acid binding protein 2; FATP4, Fatty acid transport protein 4.
